# E-Synthesis: A Bayesian Framework for Causal Assessment in Pharmacosurveillance

**DOI:** 10.3389/fphar.2019.01317

**Published:** 2019-12-17

**Authors:** Francesco De Pretis, Jürgen Landes, Barbara Osimani

**Affiliations:** ^1^Dipartimento di Scienze biomediche e Sanità pubblica, Università Politecnica delle Marche, Ancona, Italy; ^2^Dipartimento di Comunicazione ed Economia, Università degli Studi di Modena e Reggio Emilia, Reggio Emilia, Italy; ^3^Munich Center for Mathematical Philosophy, Ludwig-Maximilians-Universtät München, München, Germany

**Keywords:** adverse drug reaction, drug safety, causal assessment, Bradford Hill Guidelines, statistical evidence, evidence synthesis, evidence quality, pharmacovigilance

## Abstract

**Background:** Evidence suggesting adverse drug reactions often emerges unsystematically and unpredictably in form of anecdotal reports, case series and survey data. Safety trials and observational studies also provide crucial information regarding the (un-)safety of drugs. Hence, integrating multiple types of pharmacovigilance evidence is key to minimising the risks of harm.

**Methods:** In previous work, we began the development of a Bayesian framework for aggregating multiple types of evidence to assess the probability of a putative causal link between drugs and side effects. This framework arose out of a philosophical analysis of the Bradford Hill Guidelines. In this article, we expand the Bayesian framework and add “evidential modulators,” which bear on the assessment of the reliability of incoming study results. The overall framework for evidence synthesis, “E-Synthesis”, is then applied to a case study.

**Results:** Theoretically and computationally, E-Synthesis exploits coherence of partly or fully independent evidence converging towards the hypothesis of interest (or of conflicting evidence with respect to it), in order to update its posterior probability. With respect to other frameworks for evidence synthesis, our Bayesian model has the unique feature of grounding its inferential machinery on a consolidated theory of hypothesis confirmation (Bayesian epistemology), and in allowing any data from heterogeneous sources (cell-data, clinical trials, epidemiological studies), and methods (e.g., frequentist hypothesis testing, Bayesian adaptive trials, etc.) to be quantitatively integrated into the same inferential framework.

**Conclusions:** E-Synthesis is highly flexible concerning the allowed input, while at the same time relying on a consistent computational system, that is philosophically and statistically grounded. Furthermore, by introducing evidential modulators, and thereby breaking up the different dimensions of evidence (strength, relevance, reliability), E-Synthesis allows them to be explicitly tracked in updating causal hypotheses.

## Background

The United States Department of Health and Human Services reports that although medications help millions of people live longer and healthier lives, they are also the cause of approximately 280,000 hospital admissions each year and an estimated one-third of all adverse events in hospitals (US Department of Health and Human Services, Office of Disease Prevention and Health Promotion, 2014). The problem of adverse drug reactions is obviously not confined to the USA, but is a global issue (Edwards and Aronson, 2000; European Commission, 2008; Wu et al., 2010; Stausberg and Hasford, 2011). Evidence facilitating the prediction of adverse drug reactions often emerges unsystematically and unpredictably in the form of anecdotal reports, case series, and survey data, as well as more traditional sources, e.g., clinical trials (Price et al., 2014; Onakpoya et al., 2016). Recently, legislators have called for the integration of information coming from different sources when evaluating safety signals (European Parliament and the European Council: Directive 2010/84/EU; Regulation (EU) No 1235/2010; see also the 21st Century Cures Act, recently entered into force in the US). A similar call has also been issued by researchers (Cooper et al., 2005, p.249) and (Herxheimer, 2012). However, standard practices of evidence assessment are still mainly based on statistical standards that encounter significant difficulties with the integration of data emerging from observational and experimental studies at times on different species as well as from lab experiments and computer simulations. Clearly, there is increasing awareness of the need for tools that support the assessment of putative causal links between drugs and adverse reactions grounded on such heterogeneous evidence.

Indeed, the body of methodological work on post-marketing risk management *via* the aggregation of evidence is rapidly growing. The recent focus has been on various aspects of causal assessment based on heterogeneous evidence. Some examples include work on aggregating human and animal data (European Centre for Ecotoxicology and Toxicology of Chemicals (ECETOC), 2009), aggregation of spontaneous reports (Caster et al., 2017; Watson et al., 2018), Bayesian aggregation of safety trial data (Price et al., 2014) and data sets (Landes and Williamson, 2016), bringing together toxicology and epidemiology (Adami et al., 2011), retrieving but not assessing evidence Knowledge Base workgroup of the Observational Health Data Sciences and Informatics, 2017; Koutkias et al., 2017), assessing the evidential force of data in terms of reproducibility and replicability of the research (LeBel et al., 2018), grading certainty of evidence of effects in studies (Alonso-Coello et al., 2016), grading observational studies based on study design (Sanderson et al., 2007; Sterne et al., 2016; Wells et al., 2018), thematic synthesis of qualitative research, decision making (Thomas and Harden, 2008; Landes, 2018), providing probability bounds for an adverse event being drug induced in an individual (Murtas et al., 2017) in Pearl’s formal framework for causality (Pearl, 2000) and work on aggregating evidence generated by computational tools (Koutkias and Jaulent, 2015).

Much work has been devoted to the development of evidence synthesis methods testified by a growing number of (systematic) reviews and comparisons of evidence synthesis methods (Lucas et al., 2007; Greenhalgh et al., 2011; Kastner et al., 2012; Warren et al., 2012; van den Berg et al., 2013; Tricco et al., 2016a; Tricco et al., 2016b; Kastner et al., 2016; Shinkins et al., 2017). A number of studies argue that while there are many approaches and standards, it is not at all clear which is best (Greenhalgh et al., 2011; Warren et al., 2012; van den Berg et al., 2013; Kastner et al., 2016; Tricco et al., 2016a; Tricco et al., 2016b).

Traditional approaches supporting drug-licensing decisions are reviewed in (Puhan et al., 2012) and the changing roles of drug-licensing agencies in an evolving environment are described in (Ehmann et al., 2013). Closest to our approach are those that employ Bayesian statistics (Sutton and Abrams, 2001; Sutton et al., 2005).

However, the number of approaches that attempt to tackle the issues of aggregating different types of evidence to facilitate causal assessment of adverse drug reactions (assessing whether a drug causes an adverse reaction) straight on is rather small. One such approach is an epistemological framework based on Bradford Hill’s well-known guidelines (Hill, 1965), which continue to be an active area of research, e.g., see (Swaen and van Amelsvoort, 2009; Geneletti et al., 2011; Fedak et al., 2015).

Our work is rooted in the tradition that draws on statistical information and probabilsitc (in)dependence for the purpose of causal assessment. Challenges to Bayesian causal assessment have been raised by Dawid et al. (2016) among others.

This paper is a first step towards translating the philosophical approach to causal assessment of suspected adverse drug reactions of (Landes et al., 2018) towards an applicable framework. The rest of the paper is organised as follows. Next, we introduce and expand the approach of (Landes et al., 2018) and build a Bayesian network model for it. Then we apply the framework and model to a case study and conclude.

Here, we are mainly concerned with further developing the framework and how to – in principle – operationalise our approach. Delineated functional forms and some (conditional) probabilities serve only illustrative purposes. The focus is on how to determine them in principle and highlight roles and interactions of relevant concepts. Hence, significant further work is required before the framework is a ready-to-use tool.

## Methodology

E-Synthesis is a theoretical framework for causal assessment based on (Landes et al., 2018), we briefly present here its main components and integrate further dimensions of evidence.

### Aims and Scope

The framework in (Landes et al., 2018) aims to support decision making in drug regulatory agencies by providing a probability that a drug causes an adverse reaction.^1^

The hypothesis of interest is that “Drug 𝒟 causes harm *E* in population *U*.” To facilitate the inference from all the available evidence, indicators of causality are used. These indicators are based on Hill’s nine viewpoints for causal assessment (Hill, 1965).

### Bayesian Network Model

**Figure 1:**
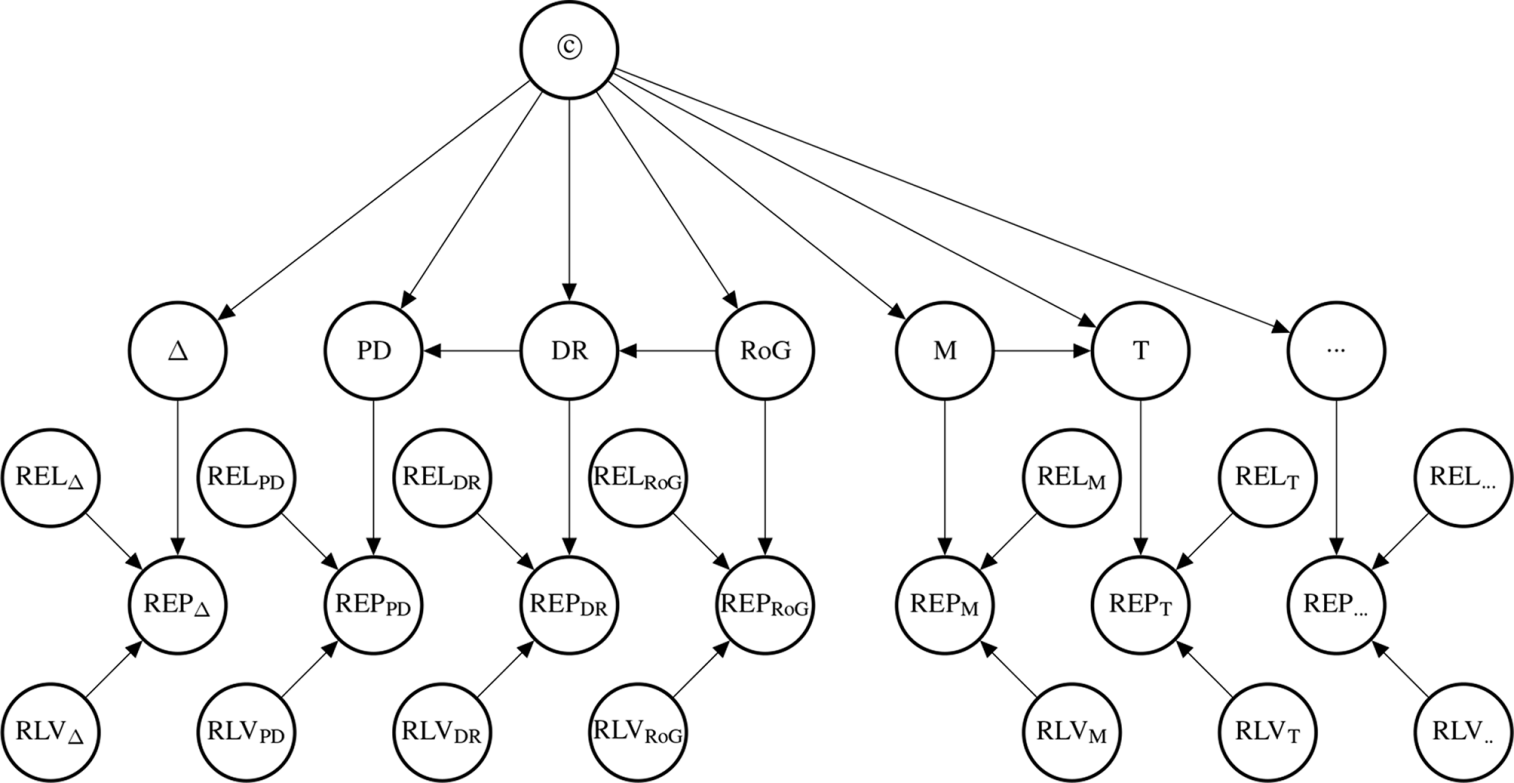
Graph of the Bayesian network with one report for every causal indicator variable taken from Landes et al. (2018). The dots indicate that there might be further indicators of causality not considered here. As explained in text, we here take it that *M* (mechanistic knowledge) entails *T* (temporal precedence) and hence introduce an arrow from *M* to *T* which is not in Landes et al. (2018). In the original paper, we considered two modulators *REL* (for “reliability” of study authors) and *RLV* [for “relevance” (external validity)] act as evidential modulators of reports (the *REP*-nodes). In this paper, we focus exclusively on the *REL* modulator which we split into a number of concepts, see *Evidential Modulators: Study Design*.

**Figure 2:**
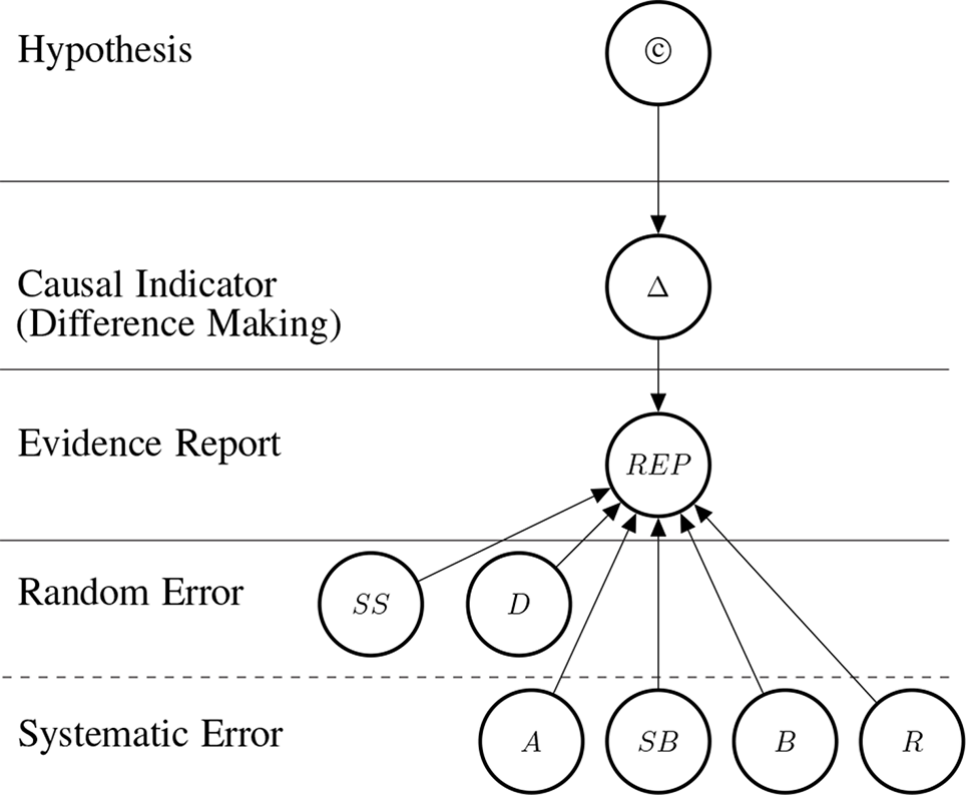
Graph structure of the Bayesian network for one RCT (randomized controlled trial) which informs us about difference making (Δ) which in turn informs us about the causal hypothesis. The information provided by the reported study is modulated by how well the particular RCT guards against random and systematic error. Duration affects systematic and random error as explained in *Control for Random Error*.

The probability of the causal hypothesis © is modelled *via* a Bayesian network of a finite number of propositional variables, see (Landes et al., 2018, *Discussion*) and see (Neapolitan, 2003) for a standard introduction to Bayesian networks. There is a binary propositional variable for the hypothesis of interest and a binary propositional variable for all six indicators of causality. For every item of evidence, every source of evidence and every study population, we create a report and evidential modulators, see **Figure 1**. The conditional probabilistic (in-)dependencies can be gleaned from the graph in terms of (Pearl, 2000)’s *d*-separation criterion. Although, the Bayesian network is used for causal assessment, the arrows in the network are *not* causal arrows in (Pearl, 2000)’s sense – they here represent epistemic probabilistic (in-)dependencies only. **Figure 2** is an example graph with only one report. The report is the only child of many parents.

The causal hypothesis represented by © is a root node.^2^ The causal indicator are parents of report nodes which mediate causal inference from the concrete data (the reports) towards the causal hypothesis. The parents at the two bottom levels are modulators of the evidential strength of the data. These incorporate considerations about the reliability of the evidence into the assessment of the hypothesis. In particular, they take into account the possibility of random error [as a function of sample size (*SS*) and study duration (*D*)], and systematic error; attenuated by adjustment or stratification (*A*), randomisation (*R*), blinding (*B*), placebo (*Pl*) and sponsorship bias (*SB*).

**Table 1:** Abbreviations.

Symbol	Intended Interpretation
*A*	Adjustment for Confounders
*avg*	Average
*B*	Blinding
*CI*	Confidence Interval
𝒟	Drug
*D*	Duration
*DR*	Dose-response Relationship
*ES*	Effect Size
*M*	Mechanistic Knowledge
*M* *_i_*	Mechanistic Hypothesis
NAQPI	N-acetyl-p-benzoquinone imine
OR	Odds Ratio
*PD*	Probabilistic Dependence
*Pl*	Placebo
*R*	Randomisation
*RCT*	Randomized Controlled Trial
*Rep*	Report Variable
*RoG*	Rate of Growth
*SB*	Sponsorship Bias
*SS*	Sample Size
*ST*	Signal-Tracking
*T*	Temporal Precedence
TRPA1	Transient Receptor Potential Ankyrin-1
©	Hypothesis of Causation
Δ	Difference Making
*μ* *_i_*	Mechanistic Report Variable
ℝ	Set of Real Numbers
Σ	Set of Statistical Indicators: *PD, DR* and *RoG*

This framework also allows for the incorporation of evidential modulators related to external validity [called “relevance” in (Landes et al., 2018)], however we will not treat them here for ease of exposition.^3^

A probability function consistent with the conditional independencies of the Bayesian network is selected which expresses our uncertainties in the tradition of Bayesian epistemology (Bovens and Hartmann, 2003; Howson and Urbach, 2006; Talbott, 2011). Unlike in “pure” Bayesian statistics, where one conditionalises on statistical models and hence obtains conditional probabilities mandated by the particular model (parameter), in Bayesian epistemology one may conditionalise on any proposition (or event), since probabilities are interpreted more widely as one’s uncertainties about general propositions; the Bayesian statistician Lindley is sympathetic to this approach (Lindley, 2000). In case one does conditionalise on a particular model, conditional probabilities are (virtually always) set to the probabilities of the statistical model.

While a certain degree of subjectivity is undeniable, there is a good argument to be made that some subjectivity is unavoidable in any approach to statistical/uncertain inference (Gelman and Hennig, 2017) and that Bayesian epistemology is in fact objective; or objectivity conducing; to some degree (Sprenger, 2019).^4^

### Theoretical Entities

Concepts of interest fall into two classes: i) a class of causal concepts comprising the hypothesis of interest and the indicators of causation and ii) a class of evidential concepts comprising evidential modulators and reports (data).

#### The Causal Hypothesis (©)

We are interested in determining the probability of the causal hypothesis that a drug 𝒟 causes a particular adverse effect *E* in a population *U* – given the available evidence. Although the hypothesis space could be in principle subdivided into three hypotheses: 1) 𝒟 causes *E*, 2) 𝒟 hinders *E*, and 3) 𝒟 does not cause *E*, we divide it here for simplicity’s sake into two alternative hypotheses: 1) 𝒟 causes *E* and 2) 𝒟 does not cause *E*, which consists of the disjunct of 2 and 3 above. To shorten notation, we use the symbol © in order to denote causation, such as in 𝒟©*E*, or simply ©.^5^

#### Indicators of Causation

Causal inference is mediated in the framework by “indicators of causation” in line with the Bradford Hill Guidelines for causation. As Hill puts it (Hill, 1965):

“None of my nine viewpoints can bring indisputable evidence for or against the cause-and-effect hypothesis and none can be required as a sine qua non. What they can do, with greater or less strength, is to help us make up our minds in the fundamental question – is there any other way of explaining the set of facts before us, is there any other equally, or more, likely than cause and effect?”^6^

In epistemic terms, causal indicators can be considered as observable and testable consequences of causal hypotheses, albeit non-deterministic consequences (with one exception); that is, they are more likely to be observed in the presence of a causal relationship and less likely in its absence, $P(\mathrm{Ind}|©)>P\left(\mathrm{Ind}\right)>P(\mathrm{Ind}|\bar{©})$ but they are not entailed by it.

The first indicator “difference-making,” Δ, is a perfect one, in that it entails causation. However, note that in our framework Δ is not entailed by causation. All other indicators are related only probabilistically to the hypothesis of causation, as we now explain.

##### Difference-Making (Δ)

If 𝒟 and *E* stand in a difference-making relationship, then changes in 𝒟 make a difference to *E* (while the reverse might not hold). In contrast with mere statistical measures of association, the difference-making relationship is an asymmetric one. Probabilistic dependence can go in both ways (e.g., if *Y* is probabilistically dependent on *X*, then also *X* is probabilistically dependent on *Y*); the same does not hold for difference making, which provides information about its direction. This explains why experimental evidence is considered particularly informative with respect to causation; the reason is exactly that in experiments, putative causes are intervened upon, in service of establishing whether they make a difference to the effect.^7^

Consistent with our choice of modelling “positive” causation only (that is instances of causation where X fosters rather than inhibiting Y), we shall understand this difference-making indicator as being true, if and only if the difference made is a positive one. *Mutatis mutandis*, this convention applies to the following three indicators as well.

##### Probabilistic Dependence (PD)

*PD* encodes whether 𝒟 and *E* are probabilistically dependent or not – such dependence naturally increases our belief in some underlying causal connection (as an indicator of causation; see, e.g., Reichenbach, 1956). Probabilistic dependence is an imperfect indicator of causation because neither entails the other. There are cases in which probabilistic dependence is created by confounding factors, as well as cases where two opposite effects of a single cause cancel each other out and produce a zero net effect.^8^

##### Dose-response Relationship (DR)

**Figure 3:**
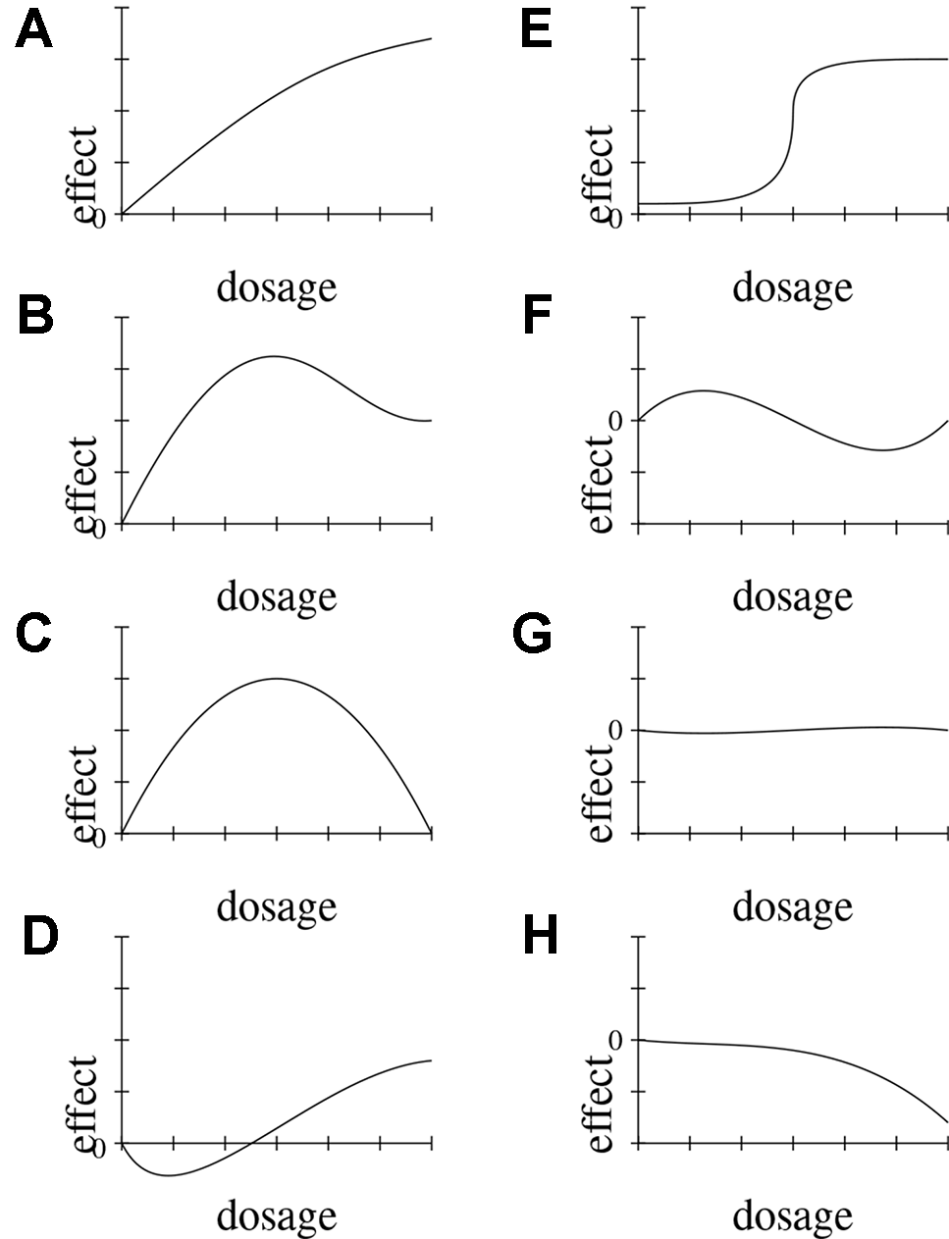
Possible functional forms of the relationship between dosage and effect. We delineate eight **(A–H)** exemplary functional forms for this relationship.

Dose-response relationships are taken as strong indicators of causation. *DR* is a stronger indicator than probabilistic dependency alone, because it requires the presence of a clear pattern of ≥3 data-points relating input and output. Indeed *DR* implies *PD*. Dose-response relationships can be inferred both at the population and at the individual level, and both in observational and experimental studies. Dose-response curves correspondingly have different scopes (e.g., the time-trend coincidence of paracetamol purchase and asthma increase in a given population [(Newson et al., 2000) vs. clinical measurements of concentration effects of analgesics]. *DR* abstracts away from these specifications and means that for dosages in the therapeutic range, the adverse effect *E* shows (approximate) monotonic growth for a significant portion of the range (see below, **Figure 3**, for an illustration of important types of dose-response curves).

##### Rate of Growth (RoG)

This indicator refers to the presence of a steep slope in the dose-response relationship. Hence, *RoG* implies a dose-response relationship (*DR* without *RoG* means either that the rate of growth is low, or highly non-linear). The indicators of causality *RoG*, *DR*, *PD* are independent of the causal structure, in the sense that they could be equally observed either in cases where 𝒟 causes *E*, or in cases where *E* causes 𝒟, or when 𝒟 and *E* have a common cause. All that matters is whether there is a (certain) systematic relationship between 𝒟 and *E*. *RoG*, *DR*, *PD* are semantically and epistemically related and we refer to them as statistical “black-box” indicators, denoted by Σ.

##### Mechanistic Knowledge (M)

*M* represents the proposition: “there is a mechanism for 𝒟 to *E*”: by which we mean a “linkage between a direct molecular initiating event [..] and an adverse outcome at a biological level of organization relevant to risk assessment” (Ankley et al., 2010, Page 731). In the biological realm, a causal relationship obviously entails the presence of a biological mechanism connecting the cause to the effect. Therefore, © ⇒ *M*. However, a mechanism may not be causally responsible for bringing out the effect due to possible inhibitors, back-up mechanisms, feedback loops, etc. *M* ⇒ © does hence not necessarily hold.

##### Time Course (T)

*T* encodes whether 𝒟 and *E* stand in the right temporal relationship (time course), which can refer to temporal order, distance, or duration. If 𝒟 causes *E*, *T* must hold (as a necessary condition): © ⇒ *T*. *T* remains an imperfect indicator, nevertheless, because temporal precedence is also compatible with ¬(𝒟 causing *E*) when 𝒟 and *E* are connected by a common cause or through reversed causation. Hence *T* ⇒ © does not necessarily hold.

#### Relationships Between Causal Indicators and the Causal Hypothesis

**Figure 4:**
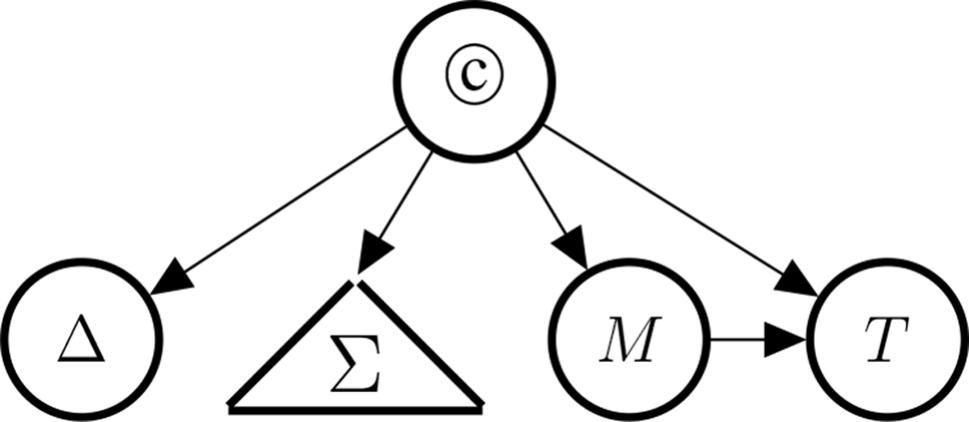
Indicators of causality, clustered by type: Δ (difference-making), Σ (the statistical blackbox) consisting of *PD* (probabilistic dependence), *DR* (dose response), and *RoG* (rate of growth), *M* (the existence of a mechanism), and *T* (temporal precedence).

As mentioned above, since they are observable consequences of ©, the causal indicators (Δ, *PD*, *DR*, *RoG*, *M*, *T*) provide support for the causal hypothesis © once concrete studies provide concrete evidence for these indicators. **Figure 4** illustrates the conceptual dependencies among these indicators (in Bayes net style). As mentioned above, not all indicators have the same strength: Δ is understood as a perfect indicator (Δ ⇒ ©) of causality. However, because of the possibility of holistic causation the inverse does not hold, that is, it is not the case that ©⇒Δ. With holistic causation we refer to cases in which causal links are embedded in a causal structure, which does not allow surgical interventions on the individual causal links (see Cartwright, 2007; Mumford and Anjum, 2011).

Landes et al. (2018) explain that although we tend to identify causation with a systematic and, possibly, asymmetric relationship between two entities or variables, yet, we prefer, in the context of causal inference, to remain neutral towards the various definitions of causation provided in terms of necessary and sufficient conditions in the philosophical literature. We choose to allow for “weaker” markers of causation, such as imperfect indicators^9^ rather than requiring the satisfaction of necessary and/or sufficient conditions of causation. However, since the indicators of causation are weaker versions of the requirements for causation formulated in the philosophical literature, this framework may be considered to generalise these requirements and the distinct ways to formulate them. A possible user is allowed to adopt (exclusively) any one of them, or to assume a pluralistic stance thereby benefiting from various inferential paths.

One reason for not having © ⇒ Δ is precisely to allow our framework to incorporate holistic conceptualisations of causation in contrast to the modular conceptualization of causes typical of the causal graph methodology developed by Pearl (2000) and Glymour and colleagues (Spirtes et al., 2000) see also Woodward (2003).^10^

The presence of a high rate of growth, *RoG*, in the dose-response relationship supports causation more strongly than the dose-response *DR* would by itself without being steep. *PD* is the weakest indicator in the Σ set. Note, however, that the statistical concepts are unrelated to difference-making information, if we have knowledge about the causal link (we will make this very fact explicit in Bayes net terms below). This reflects our intention to demarcate the conceptual divide between purely observational (symmetric) and genuinely interventional (asymmetric) indicators. Moreover, *M* entails *T* in that if there exists a mechanism linking the drug and the side effect, then it must be the case that drug administration and side effect stand in the right temporal order.

### Evolution of Our Approach

The model in (Landes et al., 2018) was developed to formalise causal inference in pharmacology on a fundamental level. It lacks the complexity necessary to capture certain important aspects of practical applications. For example, all variables are binary. Furthermore, studies are either deemed unreliable and do not provide any information whatsoever or they are deemed fully reliable and thus prove or disprove causal indicators. Conditional probabilities of causal indicators were left unspecified. Mechanistic evidence was not given particular attention.

In this paper, we allow for continuous variables taking values in the entire unit interval [0,1] ⊂ ℝ, discuss and model in detail the inferential roles of evidential modulators and thereby improve on the model of reliability (*Evidential Modulators: Study Design* and **Supplementary Material**), give a method for determining conditional probabilities of causal indicators (**Supplementary Material**) and show how mechanistic reasoning may be formalised (see Sections devoted to Mechanistic Evidence in the theoretical part and in the case study, and Section 4 of the **Supplementary Material**). In *Application of the Model: Does Paracetamol Cause Asthma?*, we show how the current model can be applied to the debated causal connection between paracetamol and asthma.

The ultimate goal is to evolve our philosophical perspectives on causal inference into a ready-to-use instrument for causal assessment supporting actual decision making procedures. This paper constitutes a step in this direction.

### Evidential Modulators: Study Design

In analogy to (Bogen and Woodward, 1988), we split the inferential path into two stages, one leading from data to abstract phenomena (here, causal indicators), and one from such phenomena to theoretical entities (here, causation). This allows us to distinguish theoretical issues related to causation and their consequences for the purpose of causal inference, from methodological concerns associated with the interpretation of data. At this second stage, we model the signal-tracking ability of the reports as a function of the instrument (the study) with which the evidence was gathered.

The signal-tracking depends on how much the study design is supposed to have controlled for systematic and random error, that is minimisation of the chances that a causal effect is wrongly attributed to the treatment under investigation, when instead the effect is due to other factors or to chance (false positive), or vice versa (false negative). Indeed, a plausible interpretation of the criterion underpinning the evidence hierarchies is the maximisation of internal validity, see also (La Caze, 2009).

However, in our view, study design also determines the kind of information that the evidence is able to provide, hence we evaluate the evidence also on the level of the kind of information it delivers: that is, the causal indicator it is able to “speak to.”

Following point 1, we associate distinct types of study design as potential carriers of causal indicators as follows:

1. Randomised Controlled Trials (RCTs) provide information about difference making, time course, possibly also dose-response relationship and rate of growth.
2. Cohort studies provide evidence of time course and statistical association (Σ).
3. Case-control studies provide information about Σ only.
4. Individual case studies cannot provide information about statistical association, but they provide very detailed information about time course and, possibly, difference-making, whenever this can be established with confidence [see for instance the Karch-Lasagna or Naranjo algorithm (Karch and Lasagna, 1977; Naranjo et al., 1981; Varallo et al., 2017)]. However, they provide very local information, about an individual subject, and therefore do not license inferences about the general population.
5. Case series can possibly help delineate a reference class, where the putative causal link holds.
6. Basic science studies (*in vitro*, or in silico), and *in vivo* studies, are generally the main source for evidence on the mechanisms underpinning the putative causal link.

The distinction of different dimensions of evidence, beyond different lines of evidence, and different inferential levels (main hypothesis, indicators, data, modulators) is the innovative point of our approach with respect to the standard view, in which these aspects are conflated, or, at least, remain implicit, in evaluating and using evidence in order to make decisions. The reason for adopting such an approach is twofold:

1. To avoid conflation of distinct ways in which the available evidence bears on the hypothesis of interest. Among others, this characterization, makes more explicit what distinguishes one method from another in terms of relevant causal information, rather than of the degree to which it avoids systematic error;
2. In our framework, evidence supports the causal indicators which in turn support the causal hypothesis of interest, each to a different degree. Hence, downgrading the evidential value of studies that feed into the weaker indicators just because of the kind of information they cannot provide, would amount to double-downgrading such evidence. For instance, evidence coming from observational studies is uninformative with respect to the Δ indicator, but may be highly informative with respect to statistical association (Σ).

Therefore, the kind of study from which the evidence derives is directly specified by the kind of indicators to which it speaks, which be found on the right side of the conditional probability (see Section 2 in the **Supplementary Material**).^11^

Additionally, studies are weighted by their degree to which they control for systematic and random error. Control for random error is operationalised in terms of Sample Size (*SS*) and Study Duration (*D*). Control for systematic error is operationalised differently for experimental vs. observational studies. We assume that for pure observational studies, signal-tracking is limited to getting the *statistical* indicators right. We consider adjustment/stratification as relevant procedures in this respect.

Instead, for experimental studies, signal-tracking relates to getting the *causal link* right. Therefore, control for systematic error also includes attributes connected to excluding alternative causal explanation for the observed effect, such as blinding, and randomisation. In both cases we add an indication of whether the study could have been intentionally biased (because of financial interests). In the following, we discuss these evidential modulators in more detail.

In the future, we hope to analyse and incorporate further modulators such as dropouts, missing data, protocol violations, whether analysis was by intent to treat and the presence or absence of further biases into our approach. For example, regarding harm assessment, which is the focus of the present study and the main goal for developing *E-Synthesis*, sponsorship bias shifts probabilities towards reports of greater safety. (Sub-conscious) biases may instead push researchers in both directions, with a higher prevalence towards reporting more publishable results: this means statistically significant evidence and/or counter-intuitive and surprising results.

Our Bayesian model is sufficiently powerful to capture uncertainties arising from inherent difficulties in assessing the degree to which studies are controlled for systematic and random error.

#### Control for Random Error

##### Sample Size (SS)

A large sample size helps to reduce confidence in the hypothesis that an observed effect (or lack thereof) is due to chance/noise/random error. The larger the sample size, the less defeasible the inference one may draw from reported results (modulo systematic error).

##### Study Duration (D)

Most drugs produce their beneficial effects within a time horizon that is well-understood at the time of drug prescription. Instead, some adverse drug reactions, such as stroke, heart attack, and cancer, may be noted only a long time after the end of the treatment. A priori, it is not clear after how long the adverse effects will materialise. Infamous examples are the DES tragedy of causing vaginal adenocarcinoma in pubertal and adult children of treated pregnant mothers (Preston, 1988) and antipsychotic drugs causing tardive dyskinesia after years of treatment (Beasley et al., 1999).

In principle, the longer the follow up, the more likely adverse drug events will be detected. Studies with a short follow up period may thus fail to detect medium to long term effects of drugs, hence they tend to produce false negatives. A study with a short follow-up period, which does not detect an adverse effect, can only count as very weak evidence against the causal hypothesis, since the adverse reaction may occur only after the end of the follow-up period (see Vandenbroucke and Psaty, 2008). However, if the drug does not cause an adverse effect, then the study duration does obviously not influence the probability of finding it in the studied population.^12^

So, study duration affects *random* error but short studies lead to a *systematic* under-reporting of harms. This explains the position of the duration node in **Figure 2**.

#### Control for Systematic Error

While large sample sizes and long-term studies allow one to reduce one’s belief in a chance result, one has thereby *not excluded* other factors that may have caused the results. For example, consider a large study that is biased in an important respect, then – when evidence is taken at face value – one may become even surer that one has nailed down the effect size of the phenomenon of interest, erroneously so (this bias tends to become “intransigent” the larger the sample size becomes; see: Holman and Bruner, 2015). In 1998, the point was made thus: “*There is a danger that meta-analyses of observational data produce very precise but equally spurious results*” (Egger et al., 1998, p. 140). This point has recently been explored in computer simulations for aggregating evidence *via* frequentist statistics (Romero, 2016).^13^

##### Blinding, Randomisation and Placebo (B,R,Pl)

The main instruments to isolate the putative causal link 𝒟 © *E* from all other possible causal effects on *E* beyond chance. This happens because, through randomisation, one has a probabilistic guarantee (modulo random error), that the treatment and the control group are comparable with respect to all these possible additional causal influences, and therefore that the observed effect is due to the treatment and only to it, see (Fuller, 2019) for a philosophical discussion. Double blinding ensures that randomisation is not biased by the researcher in order to obtain a wishful result, or by the study subjects, through so-called placebo effects.

When the experiment has no placebo arm, or none of the drugs in the control arms are sufficiently understood, then the study cannot deliver any information about Δ (and not even about any of the Σ indicators), since evidence for these indicators draws on the observed effect difference *with* and *without* the presence of the putative cause.

In fact, if the effects of distinct *putative* causes are compared against each other, without any knowledge as to their causative status, and no absolute benchmark (i.e. *absence* of all putative cause), then such relative comparison against each other only provides information about relative difference making, that is, one is not able to establish whether e.g. 1) drug 𝒜 produces an improvement of symptoms, 2) or it is drug ℬ that worsens the situation by the same amount, 3) or else, both drug 𝒜 and ℬ have opposite effects with respect to such symptoms (the former improves them, while the latter worsens them).

There are cases that can be disambiguated though, for instance when at least one of the arms but not all of them show a dose-response relationship. In this case, the very fact that some drugs do not exhibit such dose-response relationship, and some do, is taken as a sign that the latter do contribute to *E* in some way, while the former can be taken as benchmark(s).

Even when no such disambiguation is feasible, there is still a possibility to glean some information about Δ in experimental studies, by comparing the study outcomes to the base rate incidence of the same outcome measure in the sampled population. Like many other steps in causal inference this step is fraught with risks. The more tenuous the connection between study outcome and the base rate, the more risky the step. In our framework, this risk is captured by employing different values of the evidential modulator representing the quality of the implementation of placebo control.

##### Adjustment and stratification (A)

Both in experimental and observational studies data may be adjusted for covariates both in the design and in the analysis phase. This may be done in various ways: factorial design, stratification, standardisation, multivariate regression analysis, and, more recently with the aid of Propensity Score methods (Montgomery et al., 2003; Kurth et al., 2005; Schneeweiss et al., 2009; Kahlert et al., 2017). This is an important attribute in the methodology of causal inference, which is however fraught with several diagnostic pitfalls, especially due to the requirement of “causal sufficiency” (any causal inference is invalidated, if the set of covariates on which it is based misses latent variables). Adjusting *for the right covariates*, *in a sufficient causal set* leads us to detecting non-spurious statistical associations, whereas conditioning on the wrong variables leads us astray and increases the chance of false positives and negatives, see, e.g., (McCarron et al., 2010).^14^

##### Sponsorship Bias (SB)

Evidence hierarchies are one means to order study designs in terms of the potential for suffering from systematic error, either caused by confounding or by intentional distortion of the evidence. While higher level evidence – RCTs, meta-analyses, systematic reviews of meta-analyses - is in principle less manipulable (because of blinding, randomisation, and increased accuracy through data pooling), still, well-known incentives to the distortion of evidence may arise through vested interests, and compromise the reliability of the evidence at different stages of evidence collection, interpretation and evaluation quite independently of the methodology adopted (Rising et al., 2008; Wood et al., 2008; Song et al., 2009; Krauth et al., 2013; Ioannidis, 2016). Other things being equal, a sponsored study is more likely to produce results which align with the sponsor’s interest.

One persistent bias in medical research is the sponsorship bias due to the interests of the organisations funding medical research, see, e.g., (Lundh and Bero, 2017; Lundh et al., 2017). One dramatic instance of sponsorship influencing the safety evaluation of a drug is the Vioxx disaster (Jüni et al., 2004; Horton, 2004).^15^ If a drug causes an adverse reaction, a sponsorship bias tends to hide it, and therefore makes it more likely that the study delivers no reports about adverse drug events (or reports with smaller effect sizes than the drug really induces). Furthermore, by tending to distort results in a predefined direction, bias “interacts” with random error, in the sense that systematically biased procedures, when replicated, lead to increased “artificial” accuracy: it may well be that the rate of false negatives is higher for non-sponsored studies, hence apparently paradoxically, sponsorship *bias* produces “more accurate” data when no side-effects are present in reality.^16^

Regulatory constraints on medical methodology have evolved with such sources of bias in mind, see (Teira, 2013; Teira and Reiss, 2013). However, as some have recently noted, those who intend to manipulate data find ways circumventing such regulatory constraints and trigger a race of arms characterised by epistemic asymmetry (Holman, 2015; Holman and Geislar, 2019).^17^

### Reports

This section lists three possible evidence types which may be observed with respect to causal hypotheses in medicine. A certain (statistical) measure as to the effect size, evidence regarding the possible mechanisms underpinning the “phenotypic” effect, and evidence of time course (which can only come jointly with one of the other two).^18^

#### Effect Size (ES)

The medical community has developed various popular measures of the strength of observed effects: relationships between the odds ratio, hazard ratio and the relative risk are discussed in (Stare and Maucort-Boulch, 2016; Sprenger and Stegenga, 2017).^19^

These measures all refer to the average observed effect difference in the study groups. However, other measures of causal strength refer to the systematic pattern that relates treatment and effect (dose-response relationship) and to the rate at which increase in dosage increases the observed effect (rate of growth).

#### Mechanistic Evidence (ME)

Evidence speaking for or against a mechanistic hypothesis stems from basic science or animal studies, and previously established pharmacological/biochemical knowledge. It is rarely the case that a study confirms or establishes a complete mechanism of action [“a complete and detailed understanding of each and every step in the sequence of events that leads to a toxic outcome” European Centre for Ecotoxicology and Toxicology of Chemicals (ECETOC), 2007, Page 13)] by which a drug causes an adverse reaction. Instead, mechanistic knowledge is most often acquired piecemeal: incoming evidential reports are put together to complete a mechanistic puzzle, and they acquire their meaning only within the broader picture.

#### Time Course

Evidence of time precedence can come from experimental studies (e.g., RCTs), from cohort studies, from evidence of mechanisms, or from individual case studies (see preceding section). Longitudinal studies, may provide more data-points in time regarding the evolution of a phenomenon.^20^

## A Probabilistic Inference Model: Hypothesis Updating

We now show how one may model inferences from data to the causal hypothesis. Functional forms and concrete numbers are to be read as exemplary and can be found in the **Supplementary Material**.

### Causal Variables

#### The Causal Hypothesis

The *prior* probability of the binary variable © is one’s assessment of the probability that the drug causes the adverse effect after the hypothesis has been generated, without looking at any further evidence (only the evidence for generating the hypothesis may be taken into account here). The choice of a particular prior probability, *P*(©), is hence *via* case-by-case reasoning.

#### Causal Indicators

The conditional probabilities of a causal indicator variable, given its parent variables, measure how much different types of evidence contribute towards confirmation of the causal hypothesis of interest.

The conditional probabilities relating © to the causal indicators are relatively stable across applications because they relate a theoretical entity, ©, to abstract indicators (Σ, Δ, *M*, *T*), see also Swaen and van Amelsvoort (2009) p. 272. Determining some of these conditional probabilities (see **Figure 4**) of indicator variables is straight-forward due to their entailment relationships. We have

$$\begin{array}{ccc} P(M|©)=P(T|©)=P(\bar{∆}|\bar{©}) & = & 1\\ P(\mathrm{PD}|\mathrm{DR})=P(\mathrm{DR}|\mathrm{RoG})=P(T|M) & = & 1, \end{array}$$

that is, the probability of there being a mechanism given that there is a causal relationship between 𝒟 and *E* is one, just because © ⇒ *M*. The same holds for time course. Instead, the entailment relationship between Δ and © goes in the opposite direction: Δ ⇒ ©. Hence, $\bar{©}$ entails $\bar{∆}$ and consequently the probability of $\bar{∆}$ given is $\bar{©}$one.

Similarly, the probability of there being probabilistic dependence between 𝒟 and *E* given that there is a dose-response relationship between them is also one. For the same reason, the probability of there being a dose-response relationship given that there is a high rate of growth is one. Finally, since *M* entails *T*, the probability of *T* given *M* is one.^21^

#### Statistical Indicators

One may assign the remaining conditional probabilities of the other indicators in Σ by first determining a finite number of curves relating dosage and adverse effect, which plausibly represent the possible dose-response curves. Next, one observes which of these curves are compatible with *PD*, *DR*, *RoG*, ©. Then, one assigns prior probabilities to these curves conditional on the causal hypothesis holding or not. This suffices to compute the remaining conditional probabilities. For example, $P(\mathrm{PD}|©,\;\bar{\mathrm{DR}})$ is the probability of all curves when both *PD* and © hold but there is no dose-response relationship $\left(\bar{\mathrm{DR}}\right)$, divided by the probability of all curves where © holds but not *DR*. So, $P(\mathrm{PD}|©\bar{\mathrm{DR}})$ is equal to:

$$\frac{P(\mathrm{curves exhibiting PD},©\;\mathrm{and}\;\bar{\mathrm{DR}})}{P(\mathrm{curves exhibiting}\;©\;\mathrm{and}\;\bar{\mathrm{DR}})}$$

#### Difference Making

Since difference making is a very good indicator, we adopt “opinionated” conditional probabilities (reflecting tight relationships):

$$P(∆|©)\approx 1\;\mathrm{and}\;P(∆|\bar{©})=0.$$

The first probability equals, in essence, 1 minus the probability of holistic causation. For the purposes of calculations in our case study we here set this value equal to 1.

#### Time

Time precedence is guaranteed either by there being a mechanism that leads from 𝒟 to *E*, whether causal or not, or by there being a causal connection between 𝒟 and *E*. This is because, if there exists a mechanism from 𝒟 to *E*, then 𝒟 must be prior to *E*.^22^

Also if 𝒟 causes *E*, then 𝒟 must be prior to *E* as well. So:

$$P(T|M,©)=P(T|M,\bar{©})=P(T|\bar{M},©)=1.$$

The probability of there being time precedence is one, whenever either *M* or © hold.

In case *M* and © are false, we have no reasons to think that *E* is prior or posterior to 𝒟. We are hence indifferent over whether there is time precedence or not. So,

$$P(T|\bar{M},\bar{©})=0.5.$$

#### Mechanisms

If a drug causes a side effect, then this must occur *via* some mechanism, so *P*(*M*|©)=1. One’s probability that there exists a physiological mechanism from drug to adverse effect, which is not causally responsible for the effect, is $P\left(M|\bar{©}\right)$. Since the probability of there being any physiological mechanism that goes from 𝒟 to *E*, even if 𝒟 does not cause *E*, is relatively high, see Howick (2011), we set this probability to 0.5.

### Evidential Variables

Each study may yield evidence for (or against) any of our causal indicators. While experimental studies yield information about difference making in addition to probabilistic dependence and time (as well as, possibly, dose-response and rate of growth), observational studies may yield information about any one of the Σ indicators only (plus, possibly, information about time). Basic science studies or animal studies (or computational methods of various kinds) may deliver information about physiological mechanisms. See section on evidential mediators and reports (*Evidential Modulators: Study Design* and *Reports*).

We formalise the notion of incoming evidence as reports confirming or (dis-) confirming any of the indicators. These are represented in the Bayesian network as variables called *Rep* (for report). These report variables as well as the modulator variables (see *Statistical Evidence for the* Σ-*Indicators*, *Evidence of Difference-Making* below) are continuous variables, which, e.g., allow for the representation of an effect size, the duration of a study in days and the quality of randomisation.

#### Statistical Evidence for the Σ-Indicators

We assume that every observational study yields information about one Σ indicator only: i.e., each *Rep* node only has one Σ parent, graphically speaking. This parent is the strongest indicator one has evidence for. For instance, a multiple-exposure study delivering information about different effect sizes in the different arms with a steep rate of growth feeds into the *RoG* indicator only. Conversely, an observational study that delivers information about the outcome of exposed vs. non-exposed subjects only, with no graded arms differentiating among diverse dosages, will feed its evidence into the weakest Σ indicator only (*PD*).

For each observational study, the values of the following variables are pertinent for the report’s conditional probability: adjustment for confounders *A*, sample size of the study *SS*, study duration *SD* and sponsorship bias *SB*.

The variables *A*, *SS* and *D* model how well a study tracks a Σ-indicator. The better the tracking the more informative a study is, the smaller the uncertainty, ceteris paribus. There may of course be other factors for a study ability to track a signal from nature that are outside of our model.

The presence of sponsorship bias instead, in the case of drug side-effects, is expected to lead to fewer reports of suspected adverse drug reactions and smaller effect sizes, i.e., side-effects tend to be concealed. The duration of a study is not a signal-tracking component in case a causal indicator does not hold, since whatever the length of the study, this will never detect a signal that nature does not send.

**Figure 5:**
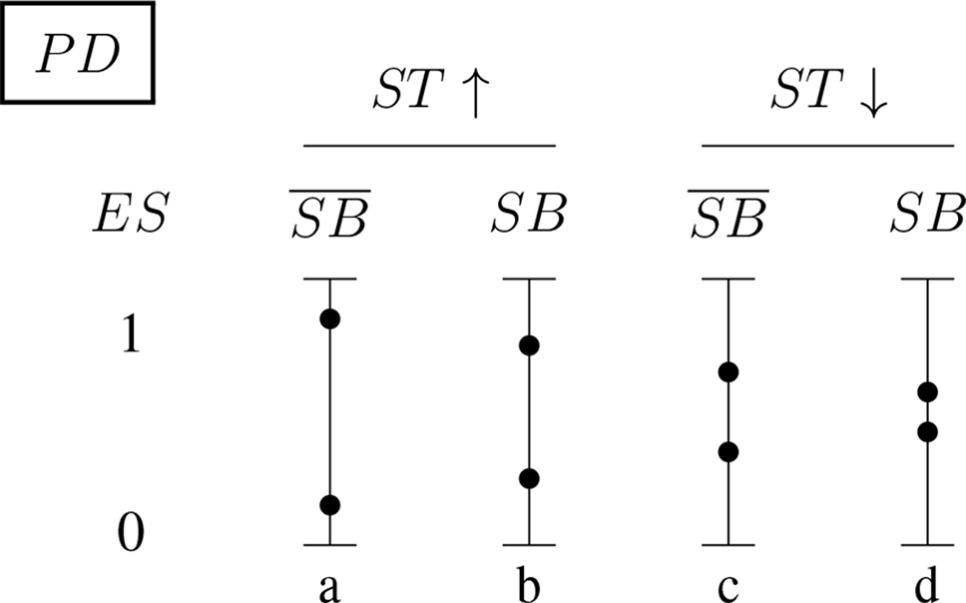
Conditional probabilities of a report (*ES* ∈ {0, 1}) for the positively instantiated *PD* (probabilistic dependence) indicator: The four bars illustrate the shift in tendency from scenario (a), with better signal-tracking and no sponsorship bias, to scenario (d), with worse signal-tracking and sponsorship bias. The probability of a report of significant effect size, *ES* = 1, decreases from left to right.

**Figure 6:**
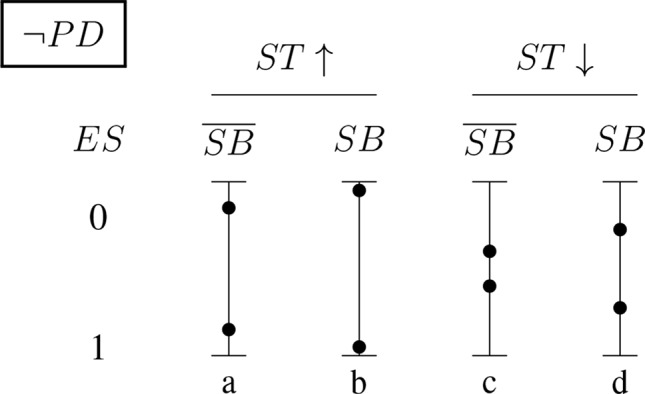
Conditional probabilities of a report (*ES* ∈ {0, 1}) for the negatively instantiated *PD* (probabilistic dependence) indicator: As above, the four bars illustrate the shift in tendency from scenario (a), with better signal-tracking and no sponsoring bias, to scenario (d), with worse signal-tracking and sponsorship bias. In this case, the probability of a report of significant effect size, *ES* = 1, increases with worsening signal-tracking, but decreases when sponsorship bias is accounted for.

**Figures 5** and **6** (for positively and negatively instantiated *PD*, respectively) compactly illustrate these shifting tendencies when these dimensions interact. The graphs show for a (non-)significant effect size, *ES* ∈ {0, 1}, how the conditional probability of a report changes (in tendency) when the sponsorship bias variable *SB* and the signal-tracking (as a composite variable) change. Case (a) represents a better signal-tracking and no sponsorship bias, case (d) represents a worse signal-tracking and the presence of sponsorship bias, that is, the tendency to hide harmful effects. For example, for positively instantiated *PD* (**Figure 5**), adding the presence of sponsorship bias compresses the range. Worsening the signal-tracking (e.g., due to reduced sample size) also has this compression effect. Consider the case of a study which reports no adverse effect: if it is good at signal-tracking and has no sponsorship bias, then the probability of reporting such a null result is low, but it increases when sponsorship bias is present.

#### Evidence of Difference-Making

RCTs inform us about the difference-making indicator of causation and whether there is time precedence. For each study, the report’s conditional probability depends on the variables we used for statistical evidence, (adjustment, sample size, duration, sponsorship bias: *A*, *SS*, *D*, *SB*), plus: blinding *B*, randomisation *R* and placebo *Pl*. Ceteris paribus, the better blinding, randomisation and placebo implementation the better a study is at tracking the signal, or, in case no signal needs to be detected, the more it reduces the chances of false positives.

#### Assessment of Modulators

The assessment of the modulators *SS*, *D* is achieved by reading off study characteristics of published reports. There is hence no uncertainty about these variables. As a result, there is no need to explicitly represent these modulators as variables in the Bayesian network.

The other modulators may be assessed by the application of quality assessment tools (QATs). In case there is uncertainty about a particular modulator applying to a study, which may be due to disagreement between different QATs Stegenga (2014) or to lack of available data, this modulator is represented by a variable *V*. The uncertainty over *V* then leads to what Bayesian statisticians call a *hierarchical model*. Instead, for a Bayesian epistemologists the modulator variable *V* is a variable like any other and she is hence prepared to assign (conditional) probabilities to it. Technically, one specifies an unconditional probability distribution over *V* reflecting this uncertainty. In the DAG one adds an arrow starting at *V* which points to the report variable. The conditional probabilities of the report variable is then specified with respect to all the possible values of all its parents (including *V*).

If an (a group of) author(s) is responsible for multiple reports which may affected from sponsorship bias, then one creates only a single variable *V* for this (group of) author(s) which modulates all these studies. This construction allows one to reason about the sponsorship bias of the (group of) author(s) from data.

#### Mechanistic Evidence

**Figure 7:**
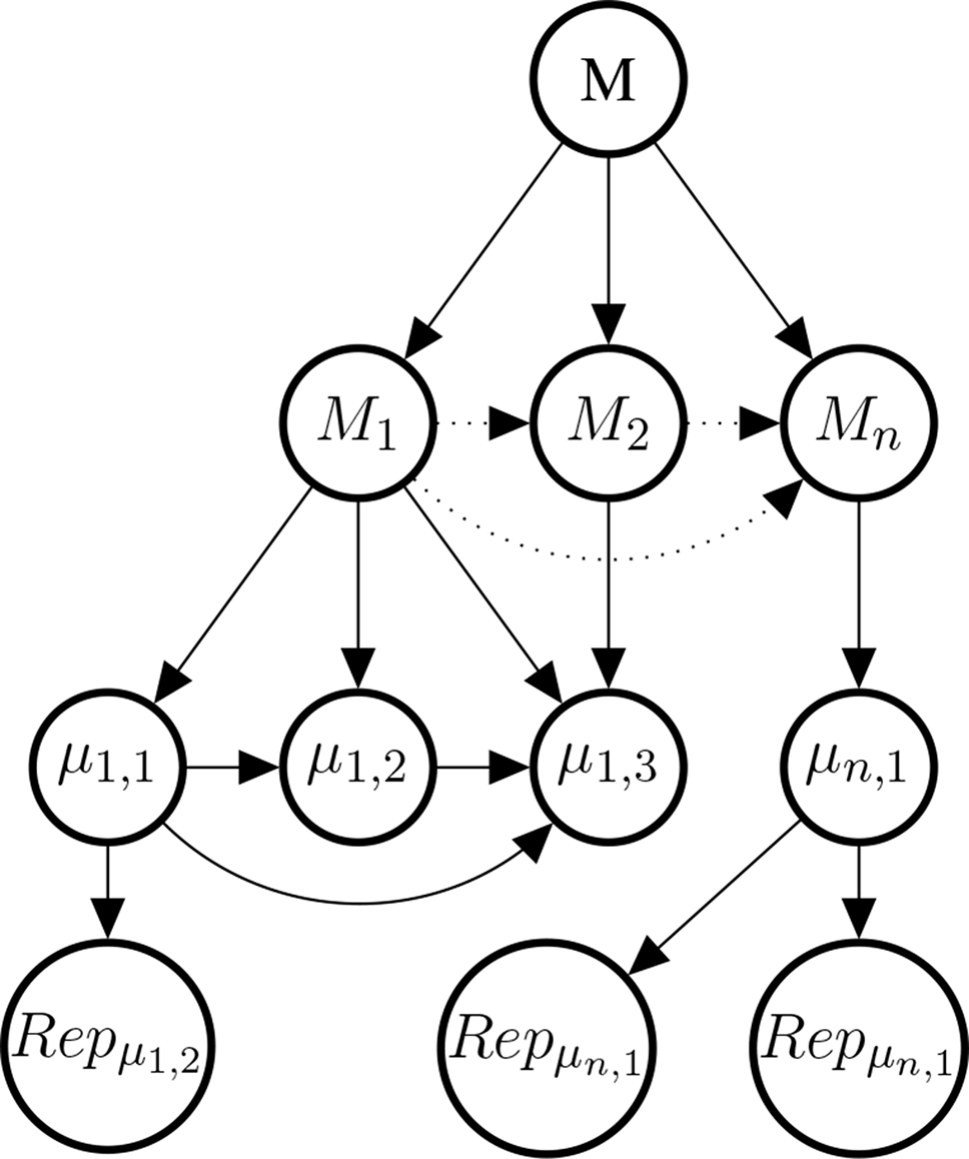
Illustrative example of the mechanism part of the Bayes net. The graph shows the existential claim *M* (mechanistic knowledge) and its relationship with hypothetical, alternative mechanisms *M*_1_,*M*_2_,…, *M* *_n_*, their constitutive sub-mechanisms (*μ* *_i,k_*) and concrete evidence $\left({\mathrm{Rep}}_{\mu_{i,k}}\right)$. Dotted edges are present, if and only if two *M* *_i_* share parts of their mechanisms. Sub-mechanisms nodes (*μ* *_i,k_*) without children are to be read as hypothesised sub-mechanisms for which no evidence is available. Every sub-mechanism may have multiple evidence reports as children which may represent basic science findings in different species or cell cultures.

Studies at the genetic, molecular, or cell level are often considered to provide evidence about the mechanisms that underpin the putative phenotypic causal relation. This observation motivates our choice of introducing a variable *M* *_i_* for every mechanism for which there is evidence. The *M* *_i_* come as hypotheses about mechanisms between 𝒟 and *E*. Each mechanism *M* *_i_* may be broken down into further bits of the mechanism; denoted here by *µ* *_i,k_*. Concrete data about these bits is denoted by ${\mathrm{Rep}}_{\mu_{\mathrm{ik}}}$, see **Figure 7** for an illustration.

As for the reports feeding into the Σ set or to Δ, also the ${\mathrm{Rep}}_{\mu_{i,k}}$ reports might be modulated by evidential modulators. However, evidential modulators are of a different nature here and deserve a separate treatment. Hence, in order to keep this paper self-contained and not to complicate calculations for the case study excessively, we do not model here evidential modulators for evidence of mechanisms.

#### Evidence of the Temporal Structure

Evidence of the temporal structure comes from RCTs, and also cohort studies which can reduce the suspicion of reverse causation, but not other confounders. Modulo other confounders, a cohort study reporting an observed effect provides evidence for a statistical correlation and the temporal structure, at the same time.

## Application of the Model: Does Paracetamol Cause Asthma?

In the following, we apply our framework to a case study: the debated causal association between paracetamol and asthma. The debate is not settled yet (Heintze and Petersen, 2013; Henderson and Shaheen, 2013; Martinez-Gimeno and García-Marcos, 2013)^23^ and evidence concerning this hypothesis is by now considerably vast and varied. For simplicity, we will here consider only exemplary studies in the entire body of now available evidence, and simulate on the basis of these studies, how hypothesis updating could be modelled in our framework. We specified the causal variables and their conditional (in-)dependencies in *Theoretical Entities*. The report variables for statistical and difference-making evidence and their conditional (in-)dependencies are described in *Evidential Variables*; their conditional probabilities are specified in Section 2 of the **Supplementary Material**. How to set up the mechanistic part of the model is explained in Section 4 of the **Supplementary Material**.

Although, evidence ought to be considered always with respect to a given population of interest; we do not make any such distinction here for the sake of a compact presentation.

We here present summaries of reported results, for none of which we claim any credit.

### Hypothesis Generation

The hypothesis of a possible causal association between paracetamol intake and asthma first emerged following the observation that the “asthma epidemic” in the western population followed the same time trend of increase in paracetamol consumption.

The data on which this observation was based initially came from a study by Varner et colleagues (Varner et al., 1998). The study aimed at explaining this epidemic through the reduction of aspirin use in the same period, due to the protective properties of aspirin against asthma in virtue of its anti-inflammatory effects. Aspirin prescription declined because the drug was discovered to be associated with Reye’s syndrome (Varner et al., 1998).

The hypothesis that asthma epidemic could be explained by the drop of aspirin prescription, was however undermined by simply considering that, if it were true, then one should have observed an equal prevalence of asthma *before* aspirin was introduced into the market, and a decrease after its introduction (Shaheen et al., 2000). Since in the same study the data-points showed a coincidence in time trends not only between asthma increase and aspirin decline, but also between increase of paracetamol sales and of asthma prevalence, this led researchers to investigate the causal hypothesis that paracetamol causes asthma; see Henderson and Shaheen (2013); Osimani (2014) for more details.

However, at the time in which this hypothesis was generated, there was little belief that the household paracetamol may be causing asthma, because of a general assumption of innocuousness. Experts at the time of hypothesis generation hence had a low prior belief in © begin true. Since we do not have access to a time travel machine, we exemplary consider three plausible values of the prior probability *P*(©): 0.01,0.005 and 0.001 for illustrative purposes.

### Statistical Evidence

The statistical and mechanistic evidence presented next is a small part of all the available evidence concerning the debated causal connection between paracetamol and asthma. Studies were selected to demonstrate the workings of the model and its versatility: some of these studies are shown below, other ones are presented in the **Supplementary Material**; exhaustiveness and representativeness were not part of our study selection procedure.

To simplify exposition we here model a state in which there is no uncertainty about the modulators applying to evidential reports, that is, one is sure whether a study is properly adjusted, blinded and so on. Furthermore, we limit ourselves here to binary effect size variables *ES* ∈ {0, 1} and discrete modulator variables in {0, 0.5, 1} about which we are certain. In the **Supplementary Material** (*Methodology*), we explain how to model uncertainty about the value of modulators variables *via* Bayesian hierarchical modelling.

Lesko and Mitchell (1999) reports a practitioner-based, double-blind, clinical trial, with random assignment of paracetamol and Ibuprofen to 27,065 children, without placebo, and with a 4-week follow-up period. The aim of the study was to investigate the safety of ibuprofen, rather than paracetamol. Relevant outcomes were hospitalisation for asthma/bronchiolitis; the relative risk for ibuprofen, compared with paracetamol was 0.9 (95% CI, 0.5−1.4). Since the confidence interval for the relative risk contains 1, there is no evidence of either of the two being more or less harmful to children. With regard to a possible sponsorship bias, this study was reported to be supported by McNeil Consumer Products Company, Fort Washington, Pennsylvania.^24^ Since the study was run without placebo and for a relatively short period, the probability of observing a null effect, as in this case, is relatively high. Furthermore, the observed null effect may be due to a) neither the drug being harmful or b) both drugs being harmful. However, this latter possibility is excluded, through implicit comparison to the base-rate incidence in the overall population. Hence, we consider this study, notwithstanding its lack of placebo, to feed into the Δ indicator. In order to update our hypothesis on this evidence (*ES* = 0), we need to fully specify all conditional probabilities of observing it, when the pertinent indicator(s) hold [or not] given the evidential modulators. We assess the modulators for this study as follows: *A* = 0.5, *SS* = 1, *D* = 0, *SB* = 1, *B* = 1, *R* = 1, *Pl* = 0.5. We use $\vec{x}$ to denote the values of the pertinent modulators here and in the following formulae.^25^

$$\begin{array}{l} P(\mathrm{ES}=0|\vec{x},∆)=1-P(\mathrm{ES}=1|\vec{x},∆)\\ \;=1-(1-\frac{\mathrm{SB}}{10})\cdot (1-0.5\;\cdot ∥1-\mathrm{avg}(A,\;\mathrm{SS},\;D,\;B,\;R,\;\mathrm{Pl})∥)\\ \;=1-0.9\cdot \left(0.5+\frac{0.5+1+0+0+1+0.5}{12}\right)=\frac{13}{40}\\ P(\mathrm{ES}=0|\vec{x},\bar{∆})=1-P(\mathrm{ES}=1|\vec{x},\bar{∆})\\ \;=1-(1-\frac{\mathrm{SB}}{10})\cdot \left(0.5\cdot ||1-\mathrm{avg}(A,\;\mathrm{SS},\;B,\;R,\;\mathrm{Pl})||\right)\\ \;=1-0.9\cdot \left(0.5\cdot \left(1-\frac{0.5+1+1+1+0.5}{5}\right)\right)=\frac{91}{100}. \end{array}$$

This formula captures the idea that, if a study is good at tracking the signal, then the probability of observing the effect, given that the related statistical indicator holds, tends to 1. Instead, the worse the study is, the smaller the probability becomes. See Section 2 of the **Supplementary Material** for further details.

Shaheen et al. (2002) reports a population based longitudinal study (Avon study). Observations are reported at different times, for a minimum of 9,400 patients: pregnant women and their babies of up to 42 months. After controlling for potential confounders, frequent paracetamol use in late pregnancy (20-32 weeks), but not in early pregnancy (< 18-20 weeks), was associated with an increased risk of wheezing in the offspring at 30-42 months (adjusted odds ratio (OR) compared with no use 2.10 (95% CI 1.30 to 3.41); p = 0.003), particularly if wheezing started before 6 months (OR 2.34 (95% CI 1.24 to 4.40); p = 0.008). Assuming a causal relation, only about 1% of wheezing at 30-42 months was attributable to this exposure. Two authors of this study (SOS and RBN) report funding from the UK Department of Health. Core funding for the long term follow up of the cohort came from the Medical Research Council, the Wellcome Trust, the UK Department of Health, the Department of the Environment, DfEE, the National Institutes of Health, a variety of medical research charities and commercial sponsors, including Stirling-Winthrop who enabled the original collection of data on paracetamol use. We model this as evidence pertaining to *DR* and *T* (since only two different non-zero dosages – never, some days, most days– were reported). For the modulators we have *SS* = 1, *D* = 1, *SB* = 0, *A* = 1 and thus

$$\begin{array}{c} P(\mathrm{ES}=1|\vec{x},\;D\;R,\;T)=0.5+\frac{\mathrm{avg}(A,\;\mathrm{SS},\;D)}{2}=1\\ P(\mathrm{ES}=1|\vec{x},\;\bar{D\;R,\;T})=0.5\cdot ∥1-\mathrm{avg}(A,\;\mathrm{SS})∥=0. \end{array}$$

### Mechanistic Evidence

To focus the exposition, we only consider two possible mechanisms (*M*_1_ and *M*_2_) by which paracetamol may cause asthma.

*M*_1_: Paracetamol is metabolised to NAPQI (N-acetyl-p-benzoquinone imine) (*µ*_1,1_), NAPQI stimulates transient receptor potential ankyrin-1 (TRPA1) (*µ*_1,2_) [reported in Nassini et al. (2010)] and TRPA1 causes airway neurogenic inflammation (*µ*_1,3_) [reported in Nassini et al. (2010)].

*M*_2_: Paracetamol depletes Gluthatione (*µ*_2,1_) [reported in Micheli et al. (1994); Kourounakis et al. (1997)], low levels of Gluthatione cause oxidative stress hyperresponsiveness in the airways (*µ*_2,2_) [reported in Smith et al. (1990); Kelly (1999)].

We set the conditional probabilities of a mechanism given *M* to:

$$\begin{array}{cc} P(M_{1}|M)=0.7 & P(M_{1}|\bar{M})=0\\ P(M_{2}|M)=0.8 & P(M_{2}|\bar{M})=0. \end{array}$$

We assessed *M*_1_ and *M*_2_ to be likely, if *M* holds; *M*_1_ was assessed to be the more likely of the two. If *M* does not hold, then all *M* *_i_* have to fail to hold and are hence assigned zero probability.

We now turn to setting conditional probabilities of the *µ*_1,_ *_k_* given *M*_1_ and given $\bar{M_{1}}$ First, recall that *M* *_i_* entails *µ* *_i_*_,_ *_k_* and hence

$$\begin{array}{r} P(\mu_{1,1}|M_{1})=1\;\\ P(\mu_{1,2}|M_{1},\mu_{1,1})=1\;\\ P(\mu_{1,3}|M_{1},\mu_{1,1},\mu_{1,2})=1. \end{array}$$

*µ*_1,1_, *µ*_1,2_, *µ*_1,3_ and $\bar{M_{1}}$ are, when taken together, logically inconsistent. So,

$$P(\mu_{1,3}|\bar{M_{1}},\mu_{1,1},\mu_{1,2})=0.$$

If *M*_1_ fails to hold, then we are indifferent about *µ*_1,3_ and *µ*_1,2_ – independently of *µ*_1,1_ (respectively *µ*_1,2_).

$$\begin{array}{c} P(\mu_{1,2}|\bar{M_{1}},\mu_{1,1})=P(\mu_{1,2}|\bar{M_{1}},\bar{\mu_{1,1}})=0.5\\ P(\mu_{1,3}|\bar{M_{1}},\bar{\mu_{1,1}},\mu_{1,2})=P(\mu_{1,3}|\bar{M_{1}},\mu_{1,1},\bar{\mu_{1,2}})=P(\mu_{1,3}|\bar{M_{1}},\bar{\mu_{1,1}},\bar{\mu_{1,2}})=0.5. \end{array}$$

In general, almost all effective drugs have toxic metabolites. We here take it as established that paracetamol is metabolised to NAPQI (independently of whether *M*_1_ holds or not) and hence put

$$P(\mu_{1,1}|\bar{M_{1}})=1.$$

Conditional probabilities of considered evidence reports in (Nassini et al., 2010) for *M*_1_ are set to:

$$\begin{array}{cc} P(\mathrm{Rep}1_{\mu_{}}|\mu_{1,2})=0.91 & P(\mathrm{Rep}1_{\mu_{}}|\bar{\mu_{1,2}})=0.09\\ P(\mathrm{Rep}2_{\mu_{}}|\mu_{1,3})=0.91 & P(\mathrm{Rep}2_{\mu_{}}|\bar{\mu_{1,3}})=0.09 \end{array}$$

We take the quotient $P({\mathrm{Rep}}_{\mu_{1,2}}|\mu_{1,2})/P({\mathrm{Rep}}_{\mu_{1,2}}|\bar{\mu_{1,2}})$ to be a measure of the strength of evidence in accordance with the literature on Bayes factors. It expresses how much more (or less) likely the received evidence is under *µ* than under $\bar{\mu }$ A Bayes factor of 91/9 ≈ 10 was chosen to model confident claims in the primary literature, while a Bayes factor of 75/25 = 3 was adopted for cautious claims. Conditional probabilities of considered evidence reports for *M*_2_:

$$\begin{array}{cc} P(\mu_{2,1}|M_{2})=1 & P(\mu_{2,1}|\bar{M_{2}})=0.01\;\\ P(\mu_{2,2}|M_{2},\mu_{2,1})=1 & \\ P(\mu_{2,2}|\bar{M_{2}},\mu_{2,1})=0 & P(\mu_{2,2}|\bar{M_{2}},\bar{\mu_{2,1}})=0.5. \end{array}$$

$P(\mu_{2,2}|\bar{M_{2}},\mu_{2,1})$ is zero for the same reasons as $P(\mu_{1,3}|\bar{M_{1}},\mu_{1,1},\mu_{1,2})$ is equal to zero. Conditional probabilities of considered evidence reports (Smith et al., 1990; Micheli et al., 1994; Kourounakis et al., 1997; Kelly, 1999) are set to

$$\begin{array}{ll} P(\mathrm{Rep}1_{\mu_{2,1}}|\mu_{2,1})=0.91 & P(\mathrm{Rep}1_{\mu_{}}|\bar{\mu_{2,1}})=0.09\\ P(\mathrm{Rep}2_{\mu_{}}|\mu_{2,1})=0.91 & P(\mathrm{Rep}2_{\mu_{}}|\bar{\mu_{2,1}})=0.09\\ P(\mathrm{Rep}1_{\mu_{}}|\mu_{2,2})=0.75 & P(\mathrm{Rep}1_{\mu_{}}|\bar{\mu_{2,2}})=0.25\\ P(\mathrm{Rep}2_{\mu_{}}|\mu_{2,2})=0.91 & P(\mathrm{Rep}2_{\mu_{}}|\bar{\mu_{2,2}})=0.09. \end{array}$$

**Figure 8:**
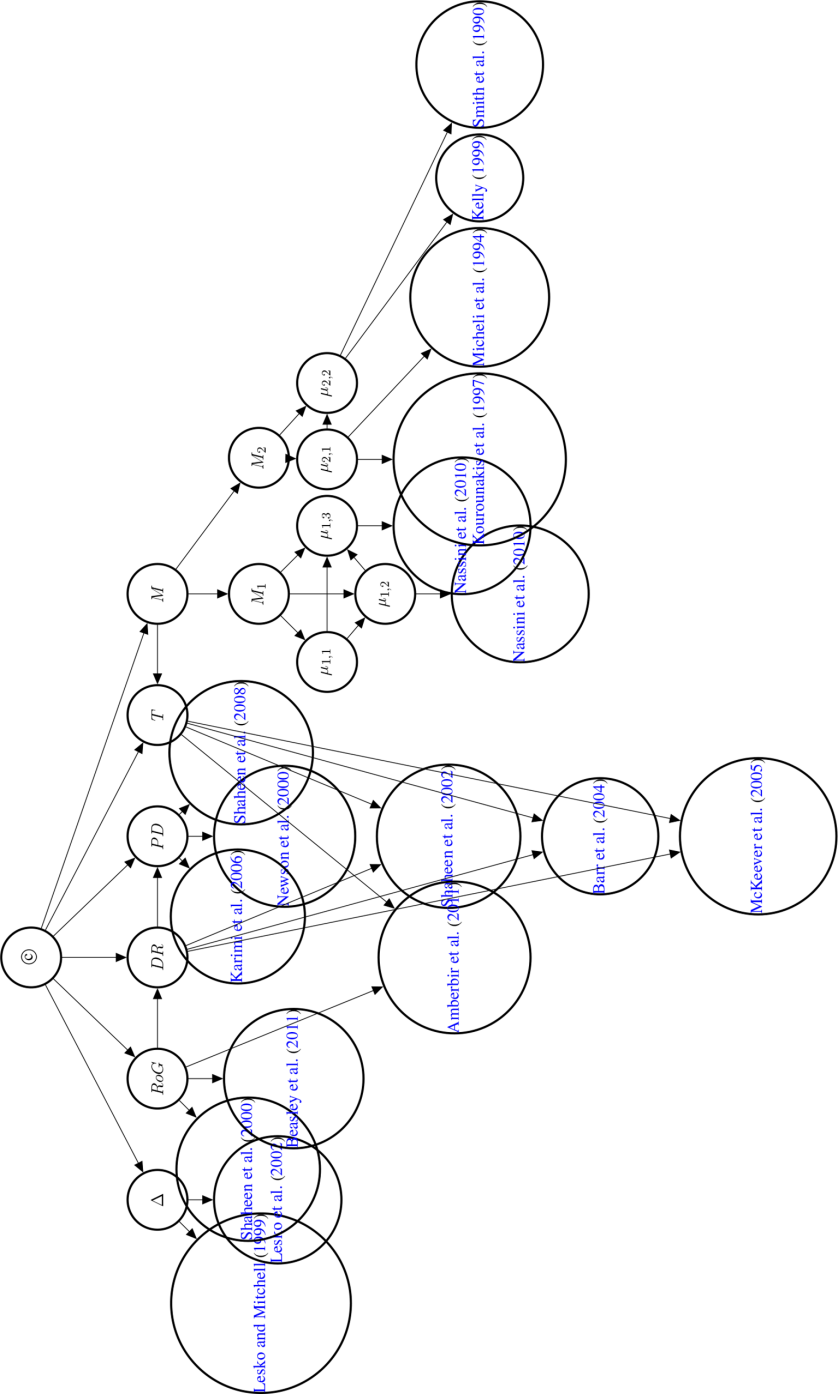
Directed acyclic graph of the Bayesian network used to compute the posterior probability of © (Hypothesis of Causation). Evidential modulators are not shown.

The first three reports are assessed as confident claims, the fourth claim as cautious. The graph of the Bayesian network is displayed in **Figure 8**.

### Posterior Probability of ©

The body of evidence incorporated here was assembled to show the versatility of the E-Synthesis framework and by no way represents a systematic review of the available evidence. The posterior probability is thus best understood as an illustration of how the framework computes the posterior without attaching too much weight to the actual computed value. We present the posteriors for the three different priors (0.01, 0.005, 0.001) as an example of a sensitivity analysis investigating the output (posterior probability of ©) on the input parameters.

We computed the posterior probability of © using the Bayes Net Toolbox in Matlab R2012a and report the posterior probabilities in **Table 2**.^26^ Formally, computing the posterior probability of © is a Bayesian network inference problem which can be solved by repeated applications of the Chain Rule and Bayes’ Theorem, see (Neapolitan, 2003).

**Table 2:** Posterior Probability of © (Hypothesis of Causation) with accumulating evidence. Every row indicates the probability of © given the body of evidence up to and including this row. Nassini et al. (2010) reports evidence for two different nodes in the Bayesian network and is hence listed twice here.

No Evidence	Prior Probability of ©	0.0100	0.0050	0.0010
Mechanistic Evidence	Smith et al. (1990)	0.0175	0.0088	0.0018
	Micheli et al. (1994)	0.0193	0.0097	0.0019
	Kourounakis et al. (1997)	0.0195	0.0098	0.0020
	Kelly (1999)	0.0196	0.0098	0.0020
	Nassini et al. (2010)a	0.0196	0.0099	0.0020
	Nassini et al. (2010)b	0.0198	0.0099	0.0020
Statistical Evidence discussed above	Lesko and Mitchell (1999)	0.0072	0.0036	0.0007
	Shaheen et al. (2002)	0.1534	0.0827	0.0176
Statistical Evidence discussed in the **Supplementary Material**	Shaheen et al. (2000)	0.2238	0.1254	0.0278
	Newson et al. (2000)	0.0997	0.0522	0.0109
	Lesko et al. (2002)	0.3686	0.2250	0.0547
	Barr et al. (2004)	0.6397	0.4690	0.1496
	McKeever et al. (2005)	0.6445	0.4742	0.1523
	Karimi et al. (2006)	0.6446	0.4743	0.1523
	Shaheen et al. (2008)	0.6446	0.4743	0.1523
	Amberbir et al. (2011)	0.7055	0.5437	0.1918
	Beasley et al. (2011)	0.7160	0.5564	0.1999
All evidence discussed here	Posterior Probability of ©	0.7160	0.5564	0.1999

## Discussion

### Learning Probabilities From Data

The (conditional) probabilities introduced above are a general example which may reflect the judgements of expert opinions. In concrete applications, these probabilities can and should heavily draw on real-world evidence and data. There is some work on how to determine such probabilities in this way, as we now briefly outline.

De Pretis and Osimani (2019) suggest an approach to compute the following:

$$P(\mathrm{DR}|{ℰ}_{\mathrm{DR}})$$

i.e., the probability of *DR*, given the available dose-response evidence and its related modulators. The MCP-Mod algorithm (see Bretz et al., 2005) and similar Bayesian approaches (Shao and Shapiro, 2019) have been proposed to tackle the problem of dose-finding in pre-clinical studies aiming to determine the (optimal) *efficacy* of a drug. These algorithms consider a finite number of dose-response curves, which is similar to our approach (**Figure 3**). Unfortunately, the approach of De Pretis and Osimani (2019) does not yet involve the role of modulators, that is, they do not compute $P(\mathrm{DR}|{ℰ}_{\mathrm{DR}},\vec{x})$. We hence cannot simply incorporate their results.

Stewart et al. (2015) presents a Bayesian network approach to judge the quality of studies within the GRADE framework, see Alonso-Coello et al. (2016). “The approach also lends itself to automation, where nodes can be parameterised either using data mining software.” (Stewart et al., 2015, Page 9).

Ryan et al. (2013) determines a conditional probability of © given a statistical association. Unfortunately, we cannot use this probability here because the conditional probabilities of *PD* we require here are also conditionalised on the (non-) existence of a dose-response.

### Evidence Synthesis in Context

Theoretically and computationally, E-Synthesis exploits coherence of partly or fully independent evidence converging towards the hypothesis (or of conflicting evidence with respect to it), in order to update its posterior probability. Propagation of probabilities hence work in a totally different sense than for causal DAGs (Pearl, 2000; Spirtes et al., 2000). Probabilities reflect here epistemic uncertainty and, loci of uncertainty are made transparent in terms of articulated (conditional) probabilities, as well as graphically traceable in terms of a DAG.

With respect to other frameworks for evidence synthesis (Greenhalgh et al., 2011; Kastner et al., 2012; Warren et al., 2012; van den Berg et al., 2013; Kastner et al., 2016; Tricco et al., 2016a; Tricco et al., 2016b; Shinkins et al., 2017), our Bayesian model has the unique feature of grounding its inferential machinery on a consolidated theory of hypothesis confirmation (Bayesian epistemology), and in allowing any data from the most heterogeneous sources (cell-data, clinical trials, epidemiological studies), and methods (e.g. frequentist hypothesis testing, Bayesian adaptive trials, etc.) to be quantitatively integrated into the same inferential framework. E-Synthesis is thus at the same time highly flexible concerning the allowed input, while at the same time relying on a consistent computational system, philosophically and statistically grounded.

By introducing evidential modulators, and thereby breaking up the different dimensions of evidence (strength, relevance, reliability) E-Synthesis allows them to be explicitly tracked in the body of evidence. This makes it possible to parcel out the strength of evidence from the method with which it was obtained.^27^ With this, E-Synthesis provides a higher order perspective on evidential support by effectively embedding these various epistemic dimensions in a concrete topology.

## Conclusions

This paper focuses on inference *within one model*, rooting in *one hypothesis*, but E-Synthesis allows for going beyond the network limits and for embedding it in an even larger network to trace the hypothesis relation with other potentially concurring hypotheses. The mechanics of Bayesian epistemology are flexible enough to permit such an augmentation for the purposes of tracing further inference patterns.^28^ For simplicity’s sake we have not presented in the current paper dimensions of evidence relating to external validity and extrapolation; however the framework itself already incorporates also this sort of evidential modulators [see (Landes et al., 2018)]. We will illustrate the functioning of these modulators in a separate paper. Further limitations are that all causal indicator variables and © (in particular, there is no way to express and reason about the strength of causation in ©), conditional probabilities were not set *via* expert elicitation and the general formulae for the conditional probabilities of evidential variables (Section 2 in the **Supplementary Materials**) for illustrative purposes.

Future work may take a number of directions such as developing scoring methods learned from data (*Discussion*) and/or based on expert opinions, applications to further case studies to demonstrate the versatility of the framework, analysis and incorporation of further evidential modulators (those mentioned at the end of *Evidential Modulators: Study Design* as well as modulators of external validity), analysis and incorporation of further biases (a catalogue of biases is currently developed at the Oxford Centre for EBM), comparing E-Synthesis to other frameworks of causal assessment *via* applications to the same case study (Abdin et al., 2019) and formally modelling and incorporating (spontaneous) case reports, evidence obtained *via* text mining and/or data base search into the framework. Finally, applying non-binary report variables to capture odds ratios, relative risks and/or confidence intervals are subject to future study.

## Author Contributions

BO developed the idea of implementing Bradford-Hill criteria into an epistemic Bayesian net, identified relevant issues in the epistemology of causation as well as in the current debate on causal and statistical inference in medicine and especially in pharmacosurveillance. JL proved the mathematical soundness of the evidence aggregation tool and helped with the analysis of emerging statistical and methodological issues. FP reviewed the paper from a mathematical point of view and contributed to address *E-Synthesis* to a pharmacovigilance perspective.

## Funding

The research for this paper was funded by the European Research Council (grant 639276), the Marche Polytechnic University (Italy) and the Munich Center for Mathematical Philosophy (MCMP, Germany). JL and FP worked at the paper as 100% research fellows within the project, whereas BO is the project PI. For the final phase of writing this manuscript JL gratefully acknowledges funding from the German Research Foundation for the grant agreements LA 4093/2-1 (Evidence and Objective Bayesian Epistemology) and LA 4093/3-1 (Foundations, Applications & Theory of Inductive Logic). JL gratefully acknowledges funding from the Deutsche Forschungsgemeinschaft (DFG, German Research Foundation) - 432308570 and 405961989.

## Conflict of Interest

The authors declare that the research was conducted in the absence of any commercial or financial relationships that could be construed as a potential conflict of interest.

## Footnotes

1 Note that the framework does not aim to provide utilities of harms, nor probabilities of expected benefits, nor utilities of benefits. When utilities of harms and benefits, as well as estimation of benefits are provided by further means, a drug regulatory agency can perform an expected utility calculation to determine whether the expected advantages of a drug exceed the expected dis-advantages. The agency will withdraw the drug (or not approve it), if the expected disadvantages outweigh the expected benefits, see (Landes et al., 2018, *Bayesian Network Model*).

2 The abbreviations used in this article are listed in **Table 1**.

3 They are extensively addressed in (Poellinger, 2019) and will be the focus of a rejoinder to this paper.

4 What probabilities at a population level may mean to an individual has recently been explored in (Dawid, 2017).

5 We do not commit here to any specific view or definition of causation [e.g., dispositional, probabilistic, counterfactual, manipulationist, etc. see also (Landes et al., 2018)]. That’s a question on the ontology of causation that we leave open for the moment. Our causal hypothesis allows for the term “causes” to cover any of the current definitions of causality to the extent that the evidence used for causal inference may be made relevant to them.

6
^6^ Bradford Hill both refers to explanatory power and likelihood as reliable grounds to justify causal judgements, and presents the respective criteria as opposed to tests of significance: “No formal tests of significance can answer those questions. Such tests can, and should, remind us of the effects that the play of chance can create, and they will instruct us on the likely magnitude of those effects. Beyond that, they contribute nothing to the proof of our hypothesis.” (Hill, 1965).

7 In philosophical terms, difference-making is understood as *ideal controlled variance* along the concept of *intervention* in manipulationist theories of causation (see Landes et al., 2018 for a detailed treatment, see also Woodward, 2003 and Pearl, 2000)): *X* is called a cause of *Y* if *Y*’s value can be varied by varying *X* (possibly upon controlling for additional variables in the given situation).

8 A well-known example of this type of cancellation is Hesslow’s birth control pills case (see, e.g., Cartwright, 2001): The contraceptive (directly) causes thrombosis but simultaneously (indirectly) prevents thrombosis by preventing pregnancy which is a cause of thrombosis. (Cartwright, 2001) discusses this case as one of the pitfalls of reducing causal analysis to probabilistic methodology alone. Of course, if cancellation is suspected, one might disable certain preventative causal routes to check whether the causal relationship actually shows once disabling conditions are held fixed. Cartwright however discusses cases where this strategy might not even be viable, owing to the complexity of the causal web.

9 That is, indicators that merely make the probability of a causal relationship being present more probable than not.

10 These approaches are under attack for failing to recognize that causal structures may be holistic (that is, synergistic and non-additive vs. modular and additive), and therefore may be not adequately captured by a difference making account. Strictly speaking this sort of criticism does not deny that Δ ⊃ ©, but only denies the reverse: © ⊃ Δ. However, in the causal graph literature the defining features for causality jointly entail that Δ ⇔ ©. We respect this debate by not collapsing Δ and © into a single node.

11 For instance, *P(ES | A, S, D, SB, PD, T)* is the conditional probability of observing blue whether there is an effect size *ES* or not in a cohort study; where the instantiated indicators on the right side of the conditional probability are *PD*, and theindicator for time course (*T*), since cohort studies provide information about both indicators (that is evidence for or against each of them).

12 A study with a long follow up may have measured safety (end-) points only at the end of the study or at multiple times. That’s a relevant consideration concerning study duration not yet incorporated here.

13 However, the probability of imbalance between the two groups decreases with increasing *N*. Hence, very large samples, especially with random sampling, may guard not only against random error, but also against some forms of confounding.

14 Our treatment of adjustment mirrors the discussion in the causal graph literature, where authors insist on proper adjustment for the computation of causal effects from observational data. Pearl (2000), e.g., devises his *do*-calculus precisely for this aim. Two graphical criteria (Pearl’s front-door and back-door criteria) help the researcher decide whether the observational data at hand suffice tocalculate causal effects without supplementing interventional information. If so, the probability-theoretic instruments of front-door adjustment and back-door adjustment can be applied to calculate the desired measure (see, e.g., Shrier and Platt, 2008 for an application of Pearl’s calculus in a medical setting). Despite the power of the calculus, the methodology naturally remains statistical (and Bayesian, so to speak), with all its shortcomings – e.g., the results must always be assessed relative to the prior choice of variables.

15 Other kinds of biases, may also affect the reliability of the evidential reports. These regards the interest of researchers to get career advancements, or other kinds of incentives for scientific reputation. However, we follow recent suggestions (Bero and Grundy, 2016) to clearly distinguish these from the former kind, and we model here only the former one, also for the sake of keeping the model simple, and the case study manageable. However, our framework can be easily expanded to include several sources and types of biases.

16 See also (Romero, 2016; Osimani and Landes, under review) for formal analyses of the role of bias and random error in hypothesis confirmation through replications.

17 The statistical literature also offers tools for the detection of publication bias, e.g., funnel plots (Duval and Tweedie, 2000).These relate to the representativeness of the set of available studies with respect to the population characteristics.

18 In individual case studies, time precedence is one of the main indicators of so called “token” causation, that is, actual cases of causation happening in individual patients. In this case, time precedence is not accompanied by statistical evidence of any kind, neither need it to come together with evidence about possible mechanisms of action, but rather stands alone.

19 In the philosophical literature, the strength of a causal relationship has also been conceived as stability, invariance, insensitivity, or non-contingency. (Woodward, 2003) proposes a conceptualisation of strength of causal relationships in terms of invariance: Such relationships are not distorted nor disrupted – even under interventions in the system; they invariantly propagate causal influence. This way to conceive stability pertains to the relevance dimension (see Landes et al, 2018), that is to the context-sensitivity of causation, and has therefore implications for external validity (see also: Osimani, 2019). We will not treat these aspects in the present paper.

20 Note that – under the assumption of a causal link between *D* and *E* – knowledge about the *T* indicator breaks the symmetric information provided by the *PD* indicator: E.g., if *D* is known tobe temporally prior to *E*, then the inference from (symmetric) probabilistic dependence between *D* and *E* to *E* causing *D* is not viable (*D* and *E* possessing a common cause is still a live option, though). Some philosophers of causation have made this explicit in their accounts of cause–effect relationships, see (Suppes, 1970).

21 Since all causal indicator variables are binary, and probabilities sum to one, we only need to specify one half of all conditional probabilities in the Bayesian net.

22 The fact that when 𝒟 causes *E*, in an individual or at a population level, it must come before *E*, does not obviously entail that the drug 𝒟 cannot also be taken after the event. We thank an anonymous reviewer for pointing this out to us.

23 An older reference which also gives an assessment of © along the Bradford Hill Criteria is (Farquhar et al., 2009, Page 39).

24 McNeil Consumer Products Company and Whitehall-Robins Healthcare (Madison, NJ). The Slone Epidemiology Unit has received or is currently receiving research support from the US Food and Drug Administration, the National Institutes of Health, and a number of pharmaceutical companies.

25 All conditional probabilities of evidential variables are here stated prior to normalization (see the **Supplementary Material (Section 2)** for rationales for normalization).

26 The underlying DAG is displayed in **Figure 8**. The Matlab R2012a .m-file used to compute the poterior probability can be found in the **Supplementary Material**.

27 Osimani and Landes investigate in (Osimani and Landes, under review) various concepts of reliability involved in such considerations.

28 Even if one is unenthusiastic with respect to the Bayesian approach: s/he can take our framework as a way to consistently structure the aggregation of evidence – as it is implicitly carried out in systematic reviews of quantitative and qualitative studies.
