# Supplementary material for: E-Synthesis: A Bayesian Framework for Causal Assessment in Pharmacosurveillance
Source: Front Pharmacol. 2019 Dec 17;10:1317. doi: 10.3389/fphar.2019.01317 (PMC6929659; doi:10.3389/fphar.2019.01317)
Supplement: Supplementary file 1 [file DataSheet_1.pdf]

frontiers

Supplementary Material

In this Supplementary Material, we give our rationales for setting (conditional) probabilities and exemplary formulae.

1 CAUSAL INDICATORS IN Σ

Our general strategy is to partition the hypothesis space related to the various Σ indicators and $\copyright$ by means of the possible functional forms that the relationship between $\mathcal{D}$ and E might take.

Figure 3 shows the list of plausible curves that might relate a given drug and a given (side-)effect (confined to the therapeutic range). The suggested partitioning is but one possible partitioning. We think that **Figure 3** shows the most important dose-response curve types, but we are aware that – from a different perspective or for a different purpose – one could come up with a coarser or more fine-grained partition, or argue for a different weighting scheme.

As mentioned in Section 2.7.2, we take it that the conditional probabilities relating $\copyright$ to the causal indicators are relatively stable across applications of causal assessments of putative adverse drug reactions. By studying a large set of settled causal assessments one can observe the number of times that a particular functional form was observed, given that a statistical indicator is true/false. One then obtains estimates of the relevant relative frequencies and uses these as conditional probabilities. Lacking such a large set we estimated these relative frequencies on the basis of our background knowledge. With the application of machine learning, database search as well as expert opinions we hope to improve these estimates in future work. We thank an anonymous referee to bring Ryan et al. (2013) to our attention in which provides a conditional probability of $\copyright$ given a probabilistic dependence. Unfortunately, we cannot use this probability here because the conditional probabilities of PD we require here are also conditionalised on the (non-)existence of a dose-response.

From these theoretical assumptions, we create **Table 1** of conditional probabilities of each curve, given $\copyright$ holding or not. Conditional probabilities when there is RoG are given in parentheses while probabilities without RoG are given without parenthesis.

We will explain the rationale of the compatibilities assigned to each indicator starting from the last curve – the least compatible with the hypothesis of causation – and going up to the curves which are more plausibly resulting from causal relationships in the biological realm.

Curve (h) is not compatible with any of the indicators, nor with $\copyright$. If $\mathcal{D}$ would cause E, it would do so in a preventative way. This is considered in our framework a case of $\overline{\copyright}$, and therefore incompatible with $\copyright$. Hence, the probability assigned to this curve, given that $\copyright$ holds, equals 0. Conversely, such a curve well represents a case of preventative causation, which in our model is part of the $\overline{\copyright}$ hypothesis; hence, the probability of such a curve, given $\overline{\copyright}$ is fairly high, and we assess it to: 40%.

Curve (g) is compatible with $\copyright$, when this is a case of causation with cancellation. The most famous theoretical example invented by philosophers to illustrate this possibility is represented by the net zero effect of oral contraceptive consumption on incidence of thrombosis through two perfectly counterbalanced pathways: one leading from oral contraceptive consumption to increased thrombosis incidence directly, and one leading to decrease of thrombosis incidence, through the decrease of pregnancies (which positively causes thrombosis), (see (Hesslow, 1976), where this example is advanced in order to divorce causation

from probabilistic dependence). Although theoretically possible, such cases of exact cancellations are considered to be rare, and therefore we assign a low probability to this curve, given that Ⓒ holds; 1%. Conversely, such curve represents the most paradigmatic case of no causal relationship holding between two variables, and therefore we assign it a probability of 50% given $\overline{Ⓒ}$.

Curve (f) represents an oscillatory phenomenon, which may be causative, but does not instantiate a dose-response relationship between $\mathcal{D}$ and E, neither a relationship of probabilistic dependence. We assign to this curve a low probability both in the case that Ⓒ holds (2%) and that it doesn't (3%).

Curve (e) represents an abrupt change, like the kind of phenomenon instantiated in anaphylactic shocks. This is obviously compatible with Ⓒ and PD, but is generally considered to be an exemplary case of lack of DR (or relatively independent from it). We assign to it a moderate probability in case Ⓒ holds (5%), and a low one if it doesn't (1%).

Curve (d) represents a case in which low dosages are protective while large doses are harmful. Since a significant part of the dosages in the therapeutic range are protective rather than harmful, this curve is not compatible with our understanding of positive causation nor with a positive dose-response relationship. However, depending on circumstances, there may be a positive probabilistic dependence between $\mathcal{D}$ and E due to a two-way interaction, e.g., produced by a common cause of $\mathcal{D}$ and E, see **Figure S1**. Hence, we assign some probability to this curve in case Ⓒ does not hold (3%), and zero probability in case Ⓒ holds.

Curve (c) represents a case of non-monotonic relationship, this is a case where the relationship between $\mathcal{D}$ and E is dosage-dependent, hence DR holds, and also one where PD holds, because, for any other value of $\mathcal{D}$ other than the maximal one, any value of E is different from the value that E takes at $\mathcal{D} = 0$. We assign this curve a high probability, given that Ⓒ holds. In particular, as for all other case were DR holds we split such probability in the case where RoG holds (that is the DR has a steep gradient), and where DR holds, but with a more mild gradient ($\overline{RoG}$). We take the former case to be relatively less likely in nature and so the ratio to be $5 : 2$ for a $\overline{RoG}$ curve vs. a RoG curve if Ⓒ holds. In case of $\overline{Ⓒ}$, than the odds become even stronger in favour of a $\overline{RoG}$ curve: $6 : 1$.

Analogously, for the remaining curves, a, and b, the same probabilities hold for the $\overline{RoG}$ vs. RoG curve in cases where Ⓒ does not hold. However, a and b are the most prevalent curves representing causal phenomena in the biological domain, hence although the odds between $\overline{RoG}$ and RoG curve remain the same ($5 : 2$), yet they share most of the probability distribution. We consider a to formalise causal phenomena in biology more generally and we assign it the greatest probability (70%) when Ⓒ holds, while b is assigned a probability of 21% in the same case.

With this partition of the possibility space at hand, we turn to determining the remaining conditional probabilities of our causal indicator variables in Σ.

Probabilistic Dependence

Given the parents of PD (Ⓒ, DR) and utilising the partition illustrated in **Table 1** (and visualized in **Figure 3**), we have

$$P(PD\,|\,Ⓒ, \overline{DR}) = \frac{P(e|Ⓒ)}{P(e|Ⓒ) + P(f|Ⓒ) + P(g|Ⓒ)} = \frac{5\%}{5\% + 2\% + 1\%} = \frac{5}{8} = 62.5\%,$$

$$P(PD\,|\,\overline{Ⓒ}, \overline{DR}) = \frac{P(d|\overline{Ⓒ}) + P(e|\overline{Ⓒ})}{\sum_{u\in\{d,e,f,g,h\}} P(u|\overline{Ⓒ})} = \frac{3+1}{3+1+3+50+40} = \frac{4}{97} \approx 4\%.$$

In the first equation, $\textcircled{c}, \overline{DR}$ limit the possibilities to the three curves (e), (f) and (g), of which only one option exhibits PD (e). In the second equation, $\overline{\textcircled{c}}, \overline{DR}$ limit the possibilities to (d) – (h), of which only (d) and (e) exhibits PD.

We recall that since DR entails PD that $P(PD|\textcircled{c}, DR) = 1 = P(PD|\overline{\textcircled{c}}, DR)$. We have hence determined all conditional probabilities of PD given its parents. The same kind of calculations are used to set conditional probabilities for DR and RoG.

Dose-Response

We are now also able to compute the following conditional probabilities:

$$P(DR|\textcircled{c}, RoG) = P(DR|\overline{\textcircled{c}}, RoG) = 1$$

$$>P(DR|\textcircled{c}, \overline{RoG}) = \frac{70}{73\frac{5}{7}} \approx 95\%$$

$$>P(DR|\overline{\textcircled{c}}, \overline{RoG}) = \frac{\frac{18}{7}}{99\frac{4}{7}} \approx 2.5\%.$$

Rate of Growth

The probabilities for RoG are

$$P(RoG|\textcircled{c}) = 26\frac{2}{7}\% \approx 26.3\%$$

$$>P(RoG|\overline{\textcircled{c}}) = \frac{3}{7}\% \approx 0.4\%.$$

2 REPORTS

Evidence for Σ Indicators

For a positive indicator $Ind \in \Sigma$, the effects of the modulators may be captured as follows:

$$\begin{aligned} &P(ES = 1 \,|\, A, SS, D, SB, Ind) \\ &= \int_{A,SS,D,SB} P(A = a, SS = ss, D = d, SB = sb)(1 - sb) \;\cdot (0.5 + \frac{STS(a, ss, d)}{2}) da\, dss\, dd\, dsb. \end{aligned} \quad \text{(S1)}$$

STS represents a function which captures how good a study is at signal-tracking. $P(A = a, SS = ss, D = d, SB = sb)$ is the probability that the modulators take these values for this study. Sample size and study duration can of course be gleaned from the study design and there is hence no uncertainty regarding the value of these modulators.

The probability of $P(A = a, SB = sb)$ makes it apparent that our model is a Bayesian hierarchical model in which one may specify a probability of parameters (the evidential modulator variables). This allows for representing and reasoning about the uncertainty arising in the assessment of studies, e.g., how well a study has adjusted for covariates.

In the following, we assume that the uncertainty of evidential modulator variables has been integrated out, that is we focus on fixed values of evidential modulator variables. To ease exposition and calculations even further, we assume that if sponsorship bias is present, then it reduces the probability of a positive effect size by 10%. We thus obtain the much simpler formulation:

$$\begin{aligned} P(ES = 1 \,|\, A, SS, D, SB, Ind) =& (1 - \frac{SB}{10}) \cdot \big(0.5 + \frac{STS(A, SS, D)}{2}\big) \\ =& (1 - \frac{SB}{10}) \cdot \big(1 - (0.5 \cdot (1 - w(A, SS, D)))\big), \end{aligned}$$

where w indicates some weighted sum, e.g., the standard average function.[1] Adjustment (A), sample size (SS) and study duration (D) determine the quality of signal-tracking of the study. We consider the three variables A, SS, D as equally important for signal-tracking and hence treat these three variables symmetric [all variables are assigned the same weight]. Addition was chosen as aggregation function rather than multiplication for the following reason. Using multiplication as aggregation function in STS would entail that a small but otherwise ideal study does not provide any evidence (since then $P(ES = 1|A, SS = 0, D, SB = 0, Ind) = 0.5 = P(ES = 1|A, SS = 0, SB = 0, \overline{Ind})$). Of course, other natural aggregation functions exist; they are heavily studied in Multi Attribute Utility Theory, see, e.g., Abdelaoui and Gonzalies (2009).

The possible presence of a sponsorship bias (SB) tends to hide side-effects and thus shifts probabilities towards null-effect reports. Hence, we weigh STS by a factor depending on the value of SB. In the absence of bias, the factor is equal to one and no adjustment takes place. In the presence of assessed sponsorship bias, $SB = 1$, and this translates into $1 - \frac{1}{10}$, which equals 0.9. Since this factor weights the entire STS after it, then this is reduced by 10%.

(S1) tracks the following considerations: if the study is good at tracking the signal, then the probability of observing the effect, given that the related statistical indicator holds, should tend to be 1. This is achieved by first measuring how good the study is via $w(A, SS, D)$. Suppose the study is perfect at tracking the signal then $1 - 1 = 0$, hence the study will report with probability 1 a significant effect: $ES = 1$. Instead, the worse the study is, the smaller this probability becomes. We formalise this by having the distance of STS from 1 being weighted by 0.5, and this product being subtracted from 1: this ensures that the study of the lowest possible quality is equally likely to report a significant effect, than it is to report a non-significant one.

The probability of observing a non-zero effect size of any given Σ indicator does not depend on the internal validity of the study, but just on the sample size and the study duration. This is because internal validity regards the exclusion of the alternative hypothesis for the observed effect in *causal* terms; that is, a possible confounder. In principle, the mere information about a statistical association between two variables does not require any discrimination of the possible underpinning causal structures. Hence, per se, internal validity warrant is irrelevant to the detection of statistical associations as such.

[1] At this point we make the convention that all modulator variables take values in the unit interval, $[0, 1]$. The convention is that 1 represents a maximally strong variable, e.g., $A = 1$: adjustment is perfect, $D = 1$: study duration is surely long enough to detect adverse effects, $SB = 1$: sponsorship is present. This convention also applies to the other modulator variables, B, R, Pl.

We see that the power (the probability of correctly rejecting the null hypothesis of the indicator failing to hold in the world) of an otherwise perfect study ($A = 1, D = 1, SB = 0$), is given by

$$\text{Power} = P(ES = 1|\,|\,1, SS, 1, 0, Ind) = \frac{5 + SS}{6}.$$

That is, a “small” (SS close to zero), but otherwise perfect, study has power of about $5/6$ and a “medium sized” (SS close to one half) study has power of approximately $11/12$.

The technical term “power” usually applies to the hypothesis of interest, we here apply it to an indicator of interest. This unusual terminology is due to the multi-layer structure of the Bayesian network in which evidence variables are separated from the hypothesis of interest variable by a layer of indicator variables. The same convention applies to our discussion of Type I errors and below and it is implicitly made in Section 3.1.1 when we talk about Bayes factors of mechanistic evidence variables.

In case the indicator does not hold, our formula is:

$$P(ES = 1 \,|\, A, SS, D, SB, \overline{Ind}) = (1 - \frac{SB}{10}) \cdot (0.5 - \frac{STS(A, SS)}{2}) = (1 - \frac{SB}{10}) \cdot \frac{1 - w(A, SS)}{2}.$$

That is, in case the indicator does not hold, then the probability of observing a significant effect size, through a good study should tend to 0. As a matter of fact, suppose that the study is a good one, then $||1 - w(A, SS)|| = 0$, therefore, the entire factor $(0.5 \cdot ||1 - w(A, SS)||) = 0$, hence we are certain to obtain a null effect.

Instead, suppose that the study is very bad, then $||1-w(A, SS)|| = 1$, hence, $(0.5 \cdot ||1-w(A, SS)||) = 0.5$, which represents a random probability of getting a positive or negative report: if the study is not good at tracking the signal, the probability of observing the effect would not so much depend on the indicator holding or not.

The probability of a Type I error (the probability of falsely rejecting the null hypothesis of $\overline{Ind}$) depending on the sample size for a otherwise perfect study is:

$$P(ES = 1 \,|\, 1, SS, 1, 0, \overline{Ind}) = \frac{1 - SS}{4}.$$

For example, in the absence of sponsorship bias, if all other modulators variables take the value zero, then the probability of a Type I error equals that of a Type II error which equals 0.5.

Compact presentations of these probabilities are given in **Table S2** and **Table S3**.

Evidence for Difference Making

We let for a tuple $\vec{x}$ of values of attribute variables:

$$P(ES = 1|\vec{x}, \Delta, T) = (1 - \frac{SB}{10}) \cdot (1 - 0.5 \cdot ||1 - avg(A, SS, D, B, R, Pl)||)$$

$$P(ES = 1|\vec{x}, \overline{\Delta}, T) = P(ES = 1|\vec{x}, \overline{\Delta}, \overline{T}) = (1 - \frac{SB}{10}) \cdot (0.5 \cdot ||1 - avg(A, SS, B, R, Pl)||).$$

As mentioned in the main text, signal-tracking for the Δ indicator also comprises evidential mediators related to internal validity: blinding (B), randomisation (R) and placebo (Pl). Nothing changes concerning the computation.

Note that since Δ entails ⓒ which entails T, $\Delta, \overline{T}$ is inconsistent. We hence do not need to specify conditional probabilities for $\Delta, \overline{T}$.

In case there is no difference making, $\overline{\Delta}$, whether there is time precedence between D and E makes no difference to the probability of observing a false positive in an RCT; which explains why the latter two conditional probabilities are equal: $P(ES = 1|\vec{x}, \overline{\Delta}, T) = P(ES = 1|\vec{x}, \overline{\Delta}, \overline{T})$. This means that Δ screens off the report from T – graphically speaking, the information from the report travels to T via Δ and ⓒ.

Hence, although an RCT report is informative about T, there is no edge linking it to T; see **Figure 2** for a graphical illustration.

Analogously, Δ also screens off the report from PD. This happens because, once the value of Δ is fixed, knowledge of PD does not change the probability of observing the related report. Similarly, once the value of Δ is fixed, observing a given report about it, does not change the posterior of PD (this just follows from Pearl's d-separation condition).

Mathematically, for all $\vec{x}$ it holds that

$$\begin{aligned} P(ES = 1|\Delta, PD, \vec{x}) &= P(ES = 1|\Delta, \overline{PD}, \vec{x}) \\ P(ES = 1|\overline{\Delta}, PD, \vec{x}) &= P(ES = 1|\overline{\Delta}, \overline{PD}, \vec{x}). \end{aligned}$$

In the Bayes net model, RCT report variables are hence not linked to PD.

Normalisation

Since our framework inherits the principles of Bayesian reasoning from the groundwork laid out in (Bovens and Hartmann, 2003), we must take into account the implications of this set-up when assigning concrete numbers to evidential reports. One of the virtues of our framework is that it is capable of transparently modelling various locuses of uncertainty. A noteworthy constraint is that the model ought never return a *certain* answer. To take this constraint into account, conditional probabilities of evidential reports should never be equal to zero nor one, and therefore should be chosen in the open interval $(0, 1)$. There are many reasons for this normalisation. It captures remaining random error which can never be fully controlled. If one does not normalise, then one would exclude with certainty that two studies of the same type with ideal attributes report conflicting results.

Renormalisation not only avoids the mathematical (and conceptual) trap of irreversibly "getting stuck on certainty", it can also be understood as an expression of *higher-order uncertainty*, and the related requirement to leave room for uncertainty about unconceived hypotheses at all inferential levels/steps.

Increasing the lower bound and decreasing the upper bound of the permissible range of conditional probabilities dampens the impact of incoming evidence on ⓒ's posterior probability.

Evidence for Temporal Structure

For cohort studies and $Ind \in \{PD, DR, RoG\}$ we let

$$\begin{aligned} & P(ES = 1 \mid A, SS, D, SB, Ind, T) \\ = \; & (1 - \frac{SB}{10})\big(1 - 0.5||1 - w(A, SS, D)||\big). \end{aligned}$$

For all other cases, when either Ind or T or both are 0, we let:

$$\begin{aligned} & P(ES = 1 \mid A, SS, D, SB, \overline{Ind, T}) \\ = \; & (1 - \frac{SB}{10})(0.5 \cdot ||1 - w(A, SS)||). \end{aligned}$$

This means that both indicators are equally relevant; hence one would be inclined to give to them the same mathematical role. Regarding the signal tracking characteristics of the study, these collectively weigh as much as the two indicators taken together, and since they are equally relevant in tracking the signal, they also have the same mathematical role.

Normalisation

We normalise $P(ES)$ as follows for all $\vec{x}$:

$$P_N(ES = 1|\vec{x}) = 0.5 + (P(ES = 1|\vec{x}) - 0.5) \cdot (1 - \epsilon).$$

We here let $\epsilon = 0.01$.

3 FURTHER STATISTICAL EVIDENCE FOR THE ASSESSMENT OF A POSSIBLE CAUSAL RELATIONSHIP BETWEEN PARACETAMOL-ASTHMA

In this section, we present further statistical evidence to exemplify the application of our model.

(Shaheen et al., 2000) reports a population based case-control study including 664 patients with asthma and 910 without asthma where main data sets are available for 1996-1997 (some of their data is also for 1999). After controlling for potential confounding factors, the odds ratio for asthma, compared with never users, was 1.06 (95% CI 0.77 to 1.45) in infrequent users (<monthly), 1.22 (0.87 to 1.72) in monthly users, 1.79 (1.21 to 2.65) in weekly users, and 2.38 (1.22 to 4.64) in daily users ($p(\text{trend}) = 0.0002$). The study was funded by the Department of Health. The original asthma survey was funded by the Medical Research Council. Amongst cases, increasing paracetamol use was associated with more severe disease. [2] [3]

This is a purely observational study, hence evidence modulators B, R, and Pl play no role here. We assess this study as reporting RoG with modulators: $SS = 0.5$, $D = 0.5$, $SB = 0$, $A = 1$. The conditional

[2] The authors add that frequency of aspirin use was not associated with asthma when cases as a whole were compared with controls, nor with severity of asthma amongst cases. We don't consider here this kind of implicit comparison between the pairs Treatment 1 vs. control against Treatment 2 vs. control. The kind of information and the type of inference related to this reasoning is also relevant for our purposes but we do not model here for simplicity's sake. We leave this for further research.

[3] Similarly to the previous footnote, the authors underline that frequent paracetamol use was positively associated with rhinitis, but aspirin use was not. An analogous kind of reasoning as hinted to in the previous footnote, applies here as well.

probabilities are hence

$$P(ES = 1|\vec{x}, RoG) = 1 - 0.5 + \frac{A + SS + D}{6}) = \frac{5}{6}$$
$$P(ES = 1|\vec{x}, \overline{RoG}) = 0.5 \cdot (1 - avg(A, SS)) = \frac{1}{8}.$$

Hence, the probability of observing such a steep trend in the data in a study adjusted for plausible confounders, with medium sized sample and medium duration is fairly high, *if RoG is really there*: $\frac{5}{6} \approx 0.83$. Conversely, the probability of observing such a steep trend in the same study, *RoG is not there* only amounts to $\frac{1}{8} = 12.5\%$.

(Newson et al., 2000) was an ecological study in which a positive association between paracetamol sales and asthma incidence is reported across countries. This association, however seems to disappear when an adjustment for "English speaking countries" is made. However, because no plausible explanation for such a phenomenon can be found, the evidence still stands in some way.

The only possible source of sponsorship bias is the acknowledgement that paracetamol sales data were provided by IMS-Health, London, UK.

We formalise this as evidence pertaining to PD with $ES = 0$, $SS = 1$, $D = 0.5$, $SB = 0$, $A = 1$ and

$$P(ES = 0|\vec{x}, PD) = 0.5 \cdot ||1 - avg(A, SS, D)|| = \frac{1}{12}$$
$$P(ES = 0|\vec{x}, \overline{PD}) = 1 - 0.5 \cdot ||1 - avg(A, SS)|| = 1.$$

(Lesko et al., 2002) compared paracetamol to ibuprofen. It finds better outcomes for ibuprofen. The authors are unsure whether this shows a protective effect of ibuprofen or a side-effect of paracetamol. We interpret their observations the second way.
The study was a randomised, double-blind, acetaminophen controlled clinical trial. 1879 children were followed for 4 weeks. Rates of hospitalisation for asthma did not vary significantly by antipyretic assignment; compared with children who were randomised to acetaminophen, the relative risk for children who were assigned to ibuprofen was 0.63 (95% confidence interval: 0.25–1.6). However, the risk of an outpatient visit for asthma was significantly lower in the ibuprofen group; compared with children who were randomised to acetaminophen, the relative risk for children who were assigned to ibuprofen was 0.56 (95% confidence interval: 0.34–0.95).
The Boston University Fever Study was supported by McNeil Consumer Healthcare Company (Fort Washington, PA). This work was supported by an unrestricted grant from Boots Health-care International (Nottingham, UK). The authors have also received support for research concerning pediatric ibuprofen from Whitehall-Robins Healthcare (Madison, NJ).

As an RCT, this study supports Δ with modulators set to $ES = 1$, $SS = 1$, $D = 0$, $SB = 0$,[4] $A = 0.5$, $B = 1$, $R = 1$, $Pl = 0.5$. The pertinent conditional probabilities are

$$P(ES = 1|\vec{x}, \Delta)$$
$$= (1 - 0.5 \cdot ||1 - avg(A, SS, D, B, R, Pl)||) = 0.8\overline{3}$$
$$P(ES = 1|\vec{x}, \overline{\Delta}) = 0.5 - \frac{avg(A, SS, B, R, Pl)}{2} = 0.1.$$

(Barr et al., 2004) reports a prospective cohort study for women. Between 1990 and 1996, 73,321 women were included in the analysis. Proportional hazard models included age, race, socioeconomic status, body mass index, smoking, other analgesic use, and postmenopausal hormone use. During 352,719 person-years of follow-up, 346 participants reported a new physician diagnosis of asthma meeting diagnostic criteria. Increasing frequency of acetaminophen use was positively associated with newly diagnosed asthma (p for trend $= 0.006$).

One of the authors declared to have received an unrestricted educational grant from Eisai, Inc. and Janssen Pharmaceutical, Inc. in 2000 to study Gastroesophageal Reflux Disease (GERD) and asthma; no other author declared a conflict of interest. These finding support DR and T in our model with modulators $SS = 1$, $D = 1$, $SB = 0$, $A = 1$ and conditional probabilities:

$$\begin{aligned} P(ES = 1|\vec{x}, DR, T) &= 1 \\ P(ES = 1|\vec{x}, \overline{DR, T}) &= 0. \end{aligned}$$

(McKeever et al., 2005) reports a cross-sectional longitudinal survey for the years 1998 - 2004 which included 13,492 subjects. An increased use of acetaminophen was associated with an increased prevalence of asthma in a dose-dependent manner. The odds ratio (OR) for increasing category of intake was 1.20 (95% confidence interval [CI],1.12–1.28; p value for trend < 0.001).

All authors declare no conflict of interest.

In our model, this study supports RoG and T with modulators $SS = 1$, $D = 1$, $SB = 0$, $A = 1$ and conditional probabilities

$$\begin{aligned} P(ES = 1|\vec{x}, RoG, T) &= 1 \\ P(ES = 1|\vec{x}, \overline{RoG, T}) &= 0. \end{aligned}$$

(Karimi et al., 2006) reports a survey conducted with standardised method (International Study of Asthma and Allergies in Childhood) concerning asthma, allergic rhinitis and eczema with 3000 children (6-7 years old) and 3000 teenagers. The time span between first and second questionnaires was one year. Their report states that in children aged 6-7 years taking acetaminophen in the first year of the life follows an increase in the prevalence of asthma and allergic rhinitis symptoms.

No sponsorship is reported. All authors declare not to have a conflict of interest.

[4] bias is improbable here, since the study was meant to show safety of Ibuprofen, not Paracetamol.

We model this study as supporting PD with modulators $SS = 0.5$, $D = 0$, $SB = 0$, $A = 0.5$ and conditional probabilities

$$
\begin{aligned}
P(ES = 1|\vec{x}, PD) = 0.5 + \frac{avg(A, SS, D)}{2} &= \frac{4}{6} \\
P(ES = 1|\vec{x}, \overline{PD}) &= \frac{1}{4}.
\end{aligned}
$$

(Shaheen et al., 2008) reports a case-control multi-centre study collecting data from May 2005 to May 2007. In a random effects meta-analysis, weekly use of paracetamol, compared with less frequent use, was strongly positively associated with asthma after controlling for confounders. There was no evidence for heterogeneity across centres. No association was seen between use of other analgesics and asthma. They compared 521 cases with a diagnosis of asthma and reporting of asthma symptoms within the last 12 months with 507 controls with no diagnosis. Adjustment for confounders was carried out.

Neither sponsorship nor conflicts of interests are reported.

We model this study as supporting PD with modulators $SS = 0.5$, $D = 0.5$, $SB = 0$, $A = 1$ and conditional probabilities

$$
\begin{aligned}
P(ES = 1|\vec{x}, PD) = 0.5 + \frac{0.5 + 0.5 + 1}{6} = \frac{5}{6} \\
P(ES = 1|\vec{x}, \overline{PD}) = 0.5 \cdot ||1 - \frac{0.5 + 1}{2}|| = \frac{1}{8}.
\end{aligned}
$$

(Amberbir et al., 2011) reports a longitudinal study from an Ethiopian birth cohort of 900 children with a follow-up of 3 years. Acetaminophen use was significantly associated with a dose-dependent increased risk of incident wheeze (adjusted odds ratio $= 1.88$ and 95% confidence interval $1.03 - 3.44$ for one to three tablets and 7.25 and $2.02 - 25.95$ for > 4 tablets in the past month at age 1 vs. never).

None of the authors declares to have a conflict of interest.

We model this study as supporting RoG and T with modulators $SS = 0.5$, $D = 1$, $SB = 0$, $A = 1$ and conditional probabilities

$$
\begin{aligned}
P(ES = 1|\vec{x}, RoG, T) = 0.5 + \frac{0.5 + 1 + 1}{6} = \frac{11}{12} \\
P(ES = 1|\vec{x}, \overline{RoG, T}) = 0.5 \cdot ||1 - \frac{0.5 + 1}{2}|| = \frac{1}{8}.
\end{aligned}
$$

As part of the International Study of Asthma and Allergies in Childhood (ISAAC) Phase Three (Beasley et al., 2011) reports of 13- to 14-year-old children completing written and video questionnaires to generate data on current symptoms of asthma, rhinoconjunctivitis, and eczema, and a written environmental questionnaire obtaining including questions on acetaminophen use in the past 12 months. $180,887$ 13 to 14-year-old children completed written and video questionnaires paracetamol use during the last year. In the multivariate analyses the recent use of acetaminophen was associated with an exposure-dependent increased risk of current asthma symptoms (OR, 1.43 [95%confidenceinterval, 1.33–1.53] and 2.51 [95% confidence interval, 2.33–2.70] for medium and high versus no use, respectively).

The study was reported to be sponsored by numerous companies.

We model this study as supporting RoG with modulators $SS = 1$, $D = 0.5$, $SB = 1$, $A = 1$ and conditional probabilities

$$P(ES = 1|\vec{x}, RoG) = 0.9 \cdot (0.5 + \frac{0.5 + 1 + 1}{6}) = \frac{9}{10} \cdot \frac{11}{12} = \frac{33}{40} = 0.825$$
$$P(ES = 1|\vec{x}, \overline{RoG}) = 0.45 \cdot (1 - \frac{1 + 1}{2}) = 0.$$

4 EVIDENCE ABOUT MECHANISMS

In general, there cannot be a guarantee that two different mechanistic pathways do not intersect. Even more than that, generally, biological pathways are embedded in a complex web of interactions. Hence, the M_i are in general not independent from each other and tend to form a clique (for every pair of such nodes there exists an edge connecting the nodes), in the directed acyclic graph of the Bayesian network (see **Figure 7**).

We also want to point out that the conditional probability of M_i holding given that M and T hold is equal to the conditional probability of M_i holding given that M holds since $M \Rightarrow T$. Furthermore, the conditional probability of M_i holding, given that M and $\overline{T}$ hold, is undefined, for the same reason. Finally, the conditional probability of M_i holding, given that $\overline{M}$ and T hold is equal 0, since $M_i \Rightarrow M$. The same obviously holds when also T does not hold, too. In mathematical formulas:

$$\begin{aligned} P(M_i|M, T) &= P(M_i|M), \text{ since } M \Rightarrow T \\ P(M_i|M, \overline{T}) &= \text{undefined} \\ P(M_i|\overline{M}, T) &= 0 = P(M_i|\overline{M}, \overline{T}). \end{aligned}$$

So, the M_i are conditionally independent of T given M. Hence, there are no edges connecting the M_i and T; see **Figure 8**.

We can fix some conditional probabilities of the M_i given M in full generality. If there exists no mechanism, $\overline{M}$, then no particular M_i can be true

$$P(M_i|\overline{M}) = 0 \text{ for all } i.$$

This entails that

$$\begin{aligned} P(M|M_i) &= \frac{P(M_i|M)P(M)}{P(M_i)} \\ &= \frac{P(M_i|M)P(M)}{P(M_i|M)P(M) + P(M_i|\overline{M})P(\overline{M})} \\ &= \frac{P(M_i|M)P(M)}{P(M_i|M)P(M)} = 1 \end{aligned}$$

which says that if we there exists a particular mechanism (M_i being true), then there really is a mechanism linking D and E. This means that there is no need to require that $P(M|M_i) = 1$ since this already follows from $P(M_i|\overline{M}) = 0$.

If it ever were the case that the M_i exhaust the set of all possible pathways from D to E, then $\sum_i P(M_i|M) \geq 1$ needs to hold; one of the M_i needs to be the one which makes M true. If on top

of that, the M_i are all disjoint and there can only be one mechanism causing E, then this sum becomes equal to one.

To determine conditional probabilities in case M is true, note first that we cannot use the concrete evidence we want to update on, since this evidence informs us about relationships between report variables and the M_i. Here, we are after the relations between M and the M_i. So, how likely is it to us that M_i is true in case M is true? A priori, the M_i are all equally likely given M. Pertinent medical background knowledge of (patho-)physiology influences one's probability judgements. These judgements cannot be made in generality; they are only feasible in concrete cases.

Mutatis mutandis, the same holds for the conditional probabilities of the $\mu_{i,k}$ given M_i, respectively $\overline{M_i}$. With respect to a fixed M_i, we may determine the proportion of the mechanistic pathway $\mu_{i,k}$ a part of it *covers*. In this context, *covering* may either mean spatio-temporally covering a certain portion of the distance from cause locus to effect locus, or also covering a certain portion of the information gap which must be bridged by glueing together inferential bits. Such judgements may influence the conditional probabilities on assigns to $\mu_{i,k}$ given M_i in a concrete case.

Evidential reports from basic science support the parts mechanistic hypotheses $\mu_{i,k}$. **Figure 7** illustrates the part of the Bayesian network below the M indicator: Concrete $Rep_{\mu_{i,k}}$ reports (if positive) support hypothesised part of the mechanistic pathways. Graphically speaking, evidence report nodes representing mechanistic information in the Bayesian network are children of all nodes $\mu_{i,k}$ they inform us about.

The constitutive sub-mechanisms $\mu_{i,k}$ about an M_i can be understood as pieces of a puzzle given by the respective, hypothetical M_i picture. Importantly, the $\mu_{i,k}$ are pieced together in an *additive* fashion to form a coherent story. In the Bayes net model, this must be accounted for by grouping concrete reports for a mechanistic hypothesis in a clique in the Bayesian network in the above sense. E.g., suppose that M_1 (consisting of $\mu_{1,1}$ and $\mu_{1,2}$) fails to hold, then $P(\mu_{1,1}|\overline{M_1}) > 0$ and $P(\mu_{1,1}|\overline{M_1}, \mu_{1,2}) = 0$, because $\mu_{1,1}$ and $\mu_{1,2}$ make up all of M_1. This means, $\mu_{1,1}$ and $\mu_{1,2}$ are not conditionally independent on M_1. Therefore, edges connecting all $\mu_{i,\cdot}$ are present in the graph to model this behaviour, see **Figure 7**.

5 MATLAB CODE

We here provide the source code for the Matlab calculations.

```
clear all
N = 31;
dag = zeros(N,N);
%Define nodes in the DAG
C = 1; Delta= 2; RoG = 3; DR = 4; PD=5; M=6; T=7; M1=8; M2=9;
Mu11=10; Mu12=11; Mu13=12; Mu21=13; Mu22=14;
RepMu12=15;RepMu13=16;Rep1Mu21=17;Rep2Mu21=18;Rep1Mu22=19;
Rep2Mu22=20;Lesko1999=21;Shaheen2000=22;Newson2000=23;
Shaheen2002=24;Lesko2002=25;Barr2004=26; McKeever2005=27;
Karimi2006=28;Shaheen2008=29;Amberbir2011=30;Beasley2011=31;
%Set edges in the DAG
dag(C,[Delta RoG DR PD T M]) = 1;
dag(RoG,DR) = 1;
dag(DR,PD)=1;
```

```
dag(M,[T M1 M2])=1;
dag(M1,[Mu11,Mu12,Mu13])=1;
dag(M2,[Mu21,Mu22])=1;
dag(Mu11,[Mu12,Mu13])=1;
dag(Mu12,Mu13)=1;
dag(Mu21,Mu22)=1;
dag(Mu12,RepMu12)=1;
dag(Mu13,RepMu13)=1;
dag(Mu21,[Rep1Mu21,Rep2Mu21])=1;
dag(Mu21,[Rep1Mu22,Rep2Mu22])=1;
dag(Delta,[Lesko1999,Lesko2002])=1;
dag(RoG,[Shaheen2000,Amberbir2011,Beasley2011])=1;
dag(PD,[Newson2000,Karimi2006,Shaheen2008])=1;
dag(DR,[Shaheen2002,Barr2004,McKeever2005])=1;
dag(T,[Shaheen2002,Barr2004,McKeever2005,Amberbir2011])=1;
%All variables are binary
discrete_nodes = 1:N;
node_sizes = 2*ones(1,N);
%``Create the Bayes net''
onodes = [];
bnet = mk_bnet(dag, node_sizes, 'discrete',...
discrete_nodes,'observed', onodes);
%Define conditional probability distributions
bnet.CPD{C} = tabular_CPD(bnet, C, [0.999 0.001]); %[P(Not C) P(C)]
bnet.CPD{Delta} = tabular_CPD(bnet, Delta, [1 0 0 1]);
%[P(Not Delta|Not C) P(Not Delta|C) P(Delta|Not C) P(Delta|C)]
bnet.CPD{RoG}=tabular_CPD(bnet,RoG,[0.996 0.737 0.004 0.263]);
bnet.CPD{DR}=tabular_CPD(bnet,DR,[0.975 0.05 0 0 0.025 0.95 1 1]);
%[P(Not DR|Not C Not RoG) P(Not DR|C Not RoG) P(Not DR|Not C RoG)
%P(Not DR|Not C RoG) P(DR|Not C Not RoG) P(DR|C Not RoG)
%P(DR|Not C RoG) P(DR|Not C RoG)]
bnet.CPD{PD}=tabular_CPD(bnet,PD, [0.959 0.375 0 0 0.041 0.625 1 1]);
bnet.CPD{M} = tabular_CPD(bnet, M, [0.5 0 0.5 1]);
bnet.CPD{T} = tabular_CPD(bnet, T, [0.5 0 0 0 0.5 1 1 1]);
bnet.CPD{M1} = tabular_CPD(bnet, M1,[1 0.3 0 0.7]);
bnet.CPD{M2} = tabular_CPD(bnet, M2, [1 0.2 0 0.8]);
bnet.CPD{Mu11} = tabular_CPD(bnet, Mu11,[0 0 1 1]);
bnet.CPD{Mu12} = tabular_CPD(bnet, Mu12,[0.5 0 0.5 0 0.5 1 0.5 1]);
bnet.CPD{Mu13} = tabular_CPD(bnet, Mu13,...
[0.5 0 0.5 0 0.5 0 1 0 0.5 1 0.5 1 0.5 1 0 1]);
bnet.CPD{Mu21} = tabular_CPD(bnet, Mu21,...
[0.99 0 0.01 1]);
bnet.CPD{Mu22} = tabular_CPD(bnet, Mu22,...
[0.5 0 1 0 0.5 1 0 1]);
bnet.CPD{RepMu12}= tabular_CPD(bnet, RepMu12,...
```

```
[0.9059 0.0941 0.0941 0.9059]);
bnet.CPD{RepMu13}= tabular_CPD(bnet, RepMu13,...
[0.9059 0.0941 0.0941 0.9059]);
bnet.CPD{Rep1Mu21}= tabular_CPD(bnet, Rep1Mu21,...
[0.9059 0.0941 0.0941 0.9059]);
bnet.CPD{Rep2Mu21}= tabular_CPD(bnet, Rep2Mu21,...
[0.9059 0.0941 0.0941 0.9059]);
bnet.CPD{Rep1Mu22}= tabular_CPD(bnet, Rep1Mu22,...
[0.7475 0.2525 0.2525 0.7475]);
bnet.CPD{Rep2Mu22}= tabular_CPD(bnet, Rep2Mu22,...
[0.9059 0.0941 0.0941 0.9059]);
bnet.CPD{Lesko1999} = tabular_CPD(bnet, Lesko1999,...
[0.9059  0.3267 0.0941 0.6733]);
bnet.CPD{Shaheen2000} = tabular_CPD(bnet, Shaheen2000,...
[0.87125 0.17 0.12875 0.83]);
bnet.CPD{Newson2000} = tabular_CPD(bnet, Newson2000,...
[0.99 0.0875 0.01 0.9125]);
bnet.CPD{Shaheen2002} = tabular_CPD(bnet, Shaheen2002,...
[0.99 0.99 0.99 0.01 0.01 0.01 0.01 0.99]);
bnet.CPD{Lesko2002} = tabular_CPD(bnet, Lesko2002,...
[0.83 0.104 0.17 0.896]);
bnet.CPD{Barr2004} = tabular_CPD(bnet, Barr2004,...
[0.99 0.99 0.99 0.01 0.01 0.01 0.01 0.99]);
bnet.CPD{McKeever2005} = tabular_CPD(bnet, McKeever2005,...
[0.99 0.99 0.99 0.01 0.01 0.01 0.01 0.99]);
bnet.CPD{Karimi2006} = tabular_CPD(bnet,Karimi2006,...
[0.7475 0.335 0.2525 0.665]);
bnet.CPD{Shaheen2008} = tabular_CPD(bnet,Shaheen2008,...
[0.62875 0.17 0.37125 0.83]);
bnet.CPD{Amberbir2011} = tabular_CPD(bnet,Amberbir2011,...
[0.9125 0.9125 0.9125 0.12875 0.0875 0.0875 0.0875 0.87125]);
bnet.CPD{Beasley2011} = tabular_CPD(bnet,Beasley2011,...
[0.99 0.17825 0.01 0.82175]);
%Setting up the observed evidence, ``2'' means report supporting C
evidence = cell(1,N);
evidence{RepMu12} = 2;
evidence{RepMu13} = 2;
evidence{Rep1Mu21} = 2;
evidence{Rep2Mu21} = 2;
evidence{Rep1Mu22} = 2;
evidence{Rep2Mu22} = 2;
evidence{Lesko1999}=1;
evidence{Shaheen2000}=2;
evidence{Newson2000}=1;
evidence{Shaheen2002}=2;
```

```
evidence{Lesko2002}=2;
evidence{Barr2004}=2;
evidence{McKeever2005}=2;
evidence{Karimi2006}=2;
evidence{Shaheen2008}=2;
evidence{Amberbir2011}=2;
evidence{Beasley2011}=2;
%Call a BNT Bayesian net inference engine to compute posterior of C
engine = jtree_inf_engine(bnet);
[engine, loglik] = enter_evidence(engine, evidence);
marg = marginal_nodes(engine, C);
marg.T
```

6 SUPPLEMENTARY REFERENCES, TABLES AND FIGURES

REFERENCES

Ryan P, Suchard MA, Schuemie M, Madigan D. Learning From Epidemiology: Interpreting Observational Database Studies for the Effects of Medical Products. *Statistics in Biopharmaceutical Research* **5** (2013) 170–179.

Hesslow G. Two Notes on the Probabilistic Approach to Causality. *Philosophy of Science* **43** (1976) 290–292. doi:10.2307/187270.

Abdelaoui M, Gonzalies C. Multi Attribute Theory. Bouyssou D, Dubois D, Prade H, Pirlot M, editors, *Decision-making Process* (London and Hoboken: Wiley), chap. 15 (2009), 579–616.

Bovens L, Hartmann S. *Bayesian Epistemology* (Oxford: Oxford University Press) (2003).

Shaheen SO, Sterne JAC, Songhurst CE, Burney PGJ. Frequent paracetamol use and asthma in adults. *Thorax* **55** (2000) 266–270. doi:10.1136/thorax.55.4.266.

Newson R, Shaheen S, Chinn S, Burney P. Paracetamol sales and atopic disease in children and adults: an ecological analysis. *European Respiratory Journal* **16** (2000) 817–823. Http://erj.ersjournals.com/content/16/5/817.full.pdf.

Lesko SM, Louik C, Vezina RM, Mitchell AA. Asthma Morbidity After the Short-Term Use of Ibuprofen in Children. *Pediatrics* **109** (2002) e20. doi:10.1542/peds.109.2.e20.

Barr RG, Wentowski CC, Curhan GC, Somers SC, Stampfer MJ, Schwartz J, et al. Prospective Study of Acetaminophen Use and Newly Diagnosed Asthma among Women. *American Journal of Respiratory and Critical Care Medicine* **169** (2004) 836–841. doi:10.1164/rccm.200304-596OC.

McKeever TM, Lewis SA, Smit HA, Burney P, Britton JR, Cassano PA. The Association of Acetaminophen, Aspirin, and Ibuprofen with Respiratory Disease and Lung Function. *American Journal of Respiratory and Critical Care Medicine* **171** (2005) 966–971. doi:10.1164/rccm.200409-1269OC.

Karimi M, Mirzaei M, Ahmadieh MH. Acetaminophen Use and the Symptoms of Asthma, Allergic Rhinitis and Eczema in Children. *Iranian Journal of Allergy, Asthma and Immunology* **5** (2006) 63–67.

Shaheen S, Potts J, Gnatiuc L, Makowska J, Kowalski ML, Joos G, et al. The relation between paracetamol use and asthma: a GA^{2}LEN European case-control study. *European Respiratory Journal* **32** (2008) 1231–1236. doi:10.1183/09031936.00039208.

Amberbir A, Medhin G, Alem A, Britton J, Davey G, Venn A. The role of acetaminophen and geohelminth infection on the incidence of wheeze and eczema. *American Journal of Respiratory and Critical Care Medicine* **183** (2011) 165–170. doi:10.1164/rccm.201006-0989OC.

Beasley RW, Clayton TO, Crane J, Lai CKW, Montefort SR, von Mutius E, et al. Acetaminophen use and risk of asthma, rhinoconjunctivitis, and eczema in adolescents. *American Journal of Respiratory and Critical Care Medicine* **183** (2011) 171–178. doi:10.1164/rccm.201005-0757OC.

TABLES

	PD	DR	RoG	$P(\cdot\|©)$	$P(\cdot\|\overline{©})$
a	✓	✓	(✓)	$\frac{50}{100}$ $(\frac{20}{100})$	$\frac{6}{7}\frac{1}{100}(\frac{1}{7}\frac{1}{100})$
b	✓	✓	(✓)	$\frac{15}{100}$ $(\frac{6}{100})$	$\frac{6}{7}\frac{1}{100}(\frac{1}{7}\frac{1}{100})$
c	✓	✓	(✓)	$\frac{5}{700}$ $(\frac{2}{700})$	$\frac{6}{7}\frac{1}{100}(\frac{1}{7}\frac{1}{100})$
d	✓	x	x	0	$\frac{3}{100}$
e	✓	x	x	$\frac{5}{100}$	$\frac{1}{100}$
f	x	x	x	$\frac{2}{100}$	$\frac{3}{100}$
g	x	x	x	$\frac{1}{100}$	$\frac{50}{100}$
h	x	x	x	0	$\frac{40}{100}$

Table S1. Compatibility of functional forms and causal indicators and conditional probabilities.

$(A+SS+D)/3$	0	$1/3$	$2/3$	1
$ES=1$ (true positive)	0.5	$2/3$	$5/6$	1
$ES=0$ (false negative)	0.5	$1/3$	$1/6$	0

Table S2. Probabilities of observing an effect, if PD (probabilistic dependence) is equal to 1 and SB (sponsorship bias) is equal to 0 for different values of study attributes.

$(A+SS)/2$	0	0.5	1
$ES=1$ (false positive)	0.5	0.25	0
$ES=0$ (true negative)	0.5	0.75	1

Table S3. Probabilities of observing an effect, if both PD (probabilistic dependence) and SB (sponsorship bias) are equal to 0.

FIGURES

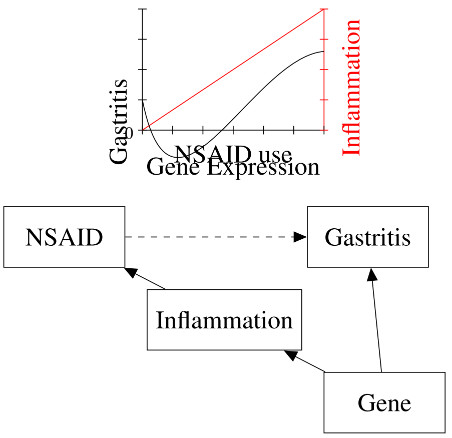

Figure S1. Bottom: Hypothetical example of a common cause structure in which a gene both causes gastritis and the use of NSAIDs (nonsteroidal anti-inflammatory drugs, via causing inflammation in the joints). The NSAID does *not* cause gastritis; however, PD (probabilistic dependence) holds between NSAID use and gastritis. It appears – prima facie – that the NSAID taken to combat the inflammation is causally influencing gastritis, whereas in actual fact gene expression is the real cause of gastritis (solid arrows do represent causal links, the dashed arrow indicates probabilistic dependence only). Top: the common cause structure in which the expression of a gene causes inflammation in a monotonic fashion (red), while gene expression in small doses protects from gastritis and no expression or too much expression of the gene do cause gastritis (black). The upshot of this state of affairs is that the hypothetically observed dose-response relationship between NSAID and gastritis is first negative and then positive and therefore incompatible with our notion of causation, whereas the probabilistic dependence is positive. Hence we have a situation where it is the case that PD and $\neg DR$ (no dose-response).
